# Atypical E3 ligase HOIL1 promotes MDA5 signaling and ester-linked ubiquitination of LGP2

**DOI:** 10.1128/mbio.02152-26

**Published:** 2026-10-22

**Authors:** Deion Cheng, Junji Zhu, W. A. Gayan Chathuranga, Surajit Chakraborty, GuanQun Liu, Rachel E. Lacoursiere, Jonathan N. Pruneda, Michaela U. Gack, Donna A. MacDuff

**Affiliations:** 1 Department of Microbiology and Immunology, University of Illinois Chicago College of Medicine 14681, Chicago, Illinois, USA; 2 Cleveland Clinic Florida Research and Innovation Center 219819, Port St. Lucie, Florida, USA; 3 Department of Molecular Microbiology and Immunology, Oregon Health & Science University 6684, Portland, Oregon, USA; Tsinghua University, Beijing, China

**Keywords:** RIG-I-like receptors, interferons, ubiquitination, LUBAC, MDA5, LGP2, HOIL1

## Abstract

The RIG-I-like receptors, RIG-I and MDA5, are innate sensors of RNA virus infections that are critical for mounting a robust antiviral immune response. Heme-oxidized IRP2 ligase 1 (HOIL1), a component of the linear ubiquitin chain assembly complex (LUBAC), is essential for interferon induction in response to viruses sensed by MDA5, but not viruses sensed by RIG-I; however, the underlying mechanism remains unclear. LUBAC contains two atypical E3 ubiquitin ligases, HOIL1 and HOIP. While HOIP generates “linear” polyubiquitin chains, HOIL1 catalyzes ester-linked ubiquitination of serine and threonine residues and glucosaccharides. Here, we show that HOIL1 E3 ligase activity is critical for MDA5-dependent interferon induction, whereas HOIP E3 ligase activity plays a minor role. Mechanistically, HOIL1 E3 ligase activity was required for MDA5 translocation to mitochondrion-associated membranes and the formation of MAVS aggregates. We further identified LGP2, a positive regulator of MDA5 oligomerization, as a HOIL1-interacting protein and substrate for ester-linked ubiquitination. In mice, loss of HOIL1 or its E3 ligase activity markedly impaired early interferon responses following infection with viruses sensed by MDA5. While HOIL1 deficiency resulted in increased viral burden and reduced survival, loss of HOIL1 ligase activity alone did not, indicating that HOIL1 exerts additional functions during antiviral immunity. Collectively, these findings establish ester-linked ubiquitination by HOIL1 as a critical mechanism regulating MDA5 signaling.

**IMPORTANCE:**

The rapid and robust production of interferon is critical for controlling viral infections. Cytoplasmic RNA sensors, RIG-I and MDA5, detect infection with distinct groups of viruses, leading to the transcriptional induction of interferon. However, inappropriate production of interferon triggered by aberrant MDA5 activation by cellular RNAs causes severe inflammatory disorders. Here, we report that the ubiquitin ligase activity of heme-oxidized IRP2 ligase 1 (HOIL1) is critical for the production of interferon in response to viruses that are sensed by MDA5 in cells and mice. We identify the third RIG-I-like receptor, LGP2, as a substrate for ester-linked ubiquitination by HOIL1, and show that, like LGP2, HOIL1 ubiquitin ligase activity is required for the efficient oligomerization of MDA5 and the initiation of downstream signaling leading to the induction of interferon. These findings reveal a new regulator of MDA5-driven interferon production and identify a potential therapeutic target for MDA5-mediated inflammatory diseases.

## INTRODUCTION

RNA virus infections are sensed by the RIG-I-like receptors (RLRs), RIG-I and MDA5, which initiate antiviral immunity through the induction of type I interferons (IFN) and inflammatory cytokines (1, 2). RIG-I recognizes short dsRNAs with 5′ tri- or di-phosphate and is the major sensor for many negative-sense RNA viruses (3, 4). In contrast, MDA5 is activated by long dsRNAs and is essential for innate sensing of coronaviruses, picornaviruses, and caliciviruses (4–7). Upon binding their respective RNA ligands, RIG-I and MDA5 oligomerize and engage the outer mitochondrial membrane protein MAVS through homotypic interactions between their caspase activation and recruitment domains (CARDs). This interaction drives MAVS aggregation into a signaling platform that recruits a number of proteins, including the transcription factors IRF3 or IRF7 and NF-κB and their activating kinases, ultimately inducing IFN, IFN-stimulated genes (ISGs), and pro-inflammatory cytokines (2, 8).

LGP2, the third RLR family member, binds RNA but lacks CARDs to initiate signaling to MAVS. Instead, LGP2 modulates activation of RIG-I and MDA5 as well as their downstream signaling (9–13). Although LGP2 can both positively and negatively regulate RLR signaling, depending in part on LGP2 expression levels (11, 13), LGP2 is crucial for MDA5 oligomer formation and activation along dsRNA (13, 14). Thus, LGP2 deficiency nearly abolishes the IFN response upon infection with viruses that are detected by MDA5 (10).

MDA5 and RIG-I activation and signaling are controlled by distinct networks of protein interactions, post-translational modifications, and other regulatory mechanisms (2) that ensure robust antiviral immunity while limiting pathological IFN and inflammatory responses (15–17). Among these regulators, heme-oxidized IRP2 ligase 1 (HOIL1; also known as RBCK1) is required for MDA5 signaling and restricts norovirus infection in mice but appears dispensable for RIG-I signaling (18). However, the functional contribution of HOIL1 remains ambiguous due to its integral role within the linear ubiquitin chain assembly complex (LUBAC), together with HOIP (RNF31) and SHARPIN. LUBAC generates methionine-1 (M1)-linked, “linear,” polyubiquitin chains (19–22) that regulate multiple immune-related pathways, including canonical NF-κB activation downstream of Toll-like receptors and various cytokine receptors, and extrinsic cell death pathways (19, 20, 23–30). LUBAC has been reported to both inhibit and promote RIG-I signaling, suggesting context-dependent roles in antiviral immunity (31–35).

Within LUBAC, HOIP is the catalytic E3 ligase responsible for M1-ubiquitination, while HOIL1 and SHARPIN stabilize the complex and allosterically activate HOIP (19–23, 36, 37). HOIL1 also possesses intrinsic E3 ligase activity that catalyzes non-canonical ester-linked ubiquitination of serine and threonine residues of proteins, including ubiquitin itself, as well as of hydroxyl groups in glucosaccharides (37–45). HOIL1-mediated ubiquitination restrains HOIP-dependent M1-polyubiquitin chain formation and NF-κB signaling (39–41, 46, 47); however, the functional outcomes of ester-linked ubiquitination catalyzed by HOIL1 in other contexts are poorly defined.

Here, we show that the E3 ligase activity of HOIL1 is necessary for MDA5 antiviral signaling, promoting MDA5 recruitment to mitochondria and subsequent MAVS aggregation and activation. Furthermore, we found that HOIL1 can function independently of its association with HOIP and SHARPIN to facilitate MDA5-mediated IFN induction. In mice, HOIL1 E3 ligase activity is required for robust IFN responses to viruses sensed by MDA5. Finally, we identified LGP2 as a substrate for HOIL1-mediated ester-linked ubiquitination, providing a mechanistic link between HOIL1’s E3 ligase activity and MDA5 activation by LGP2.

## RESULTS

### HOIL1 E3 ligase activity is critical for MDA5 antiviral signaling

**Fig 1:**
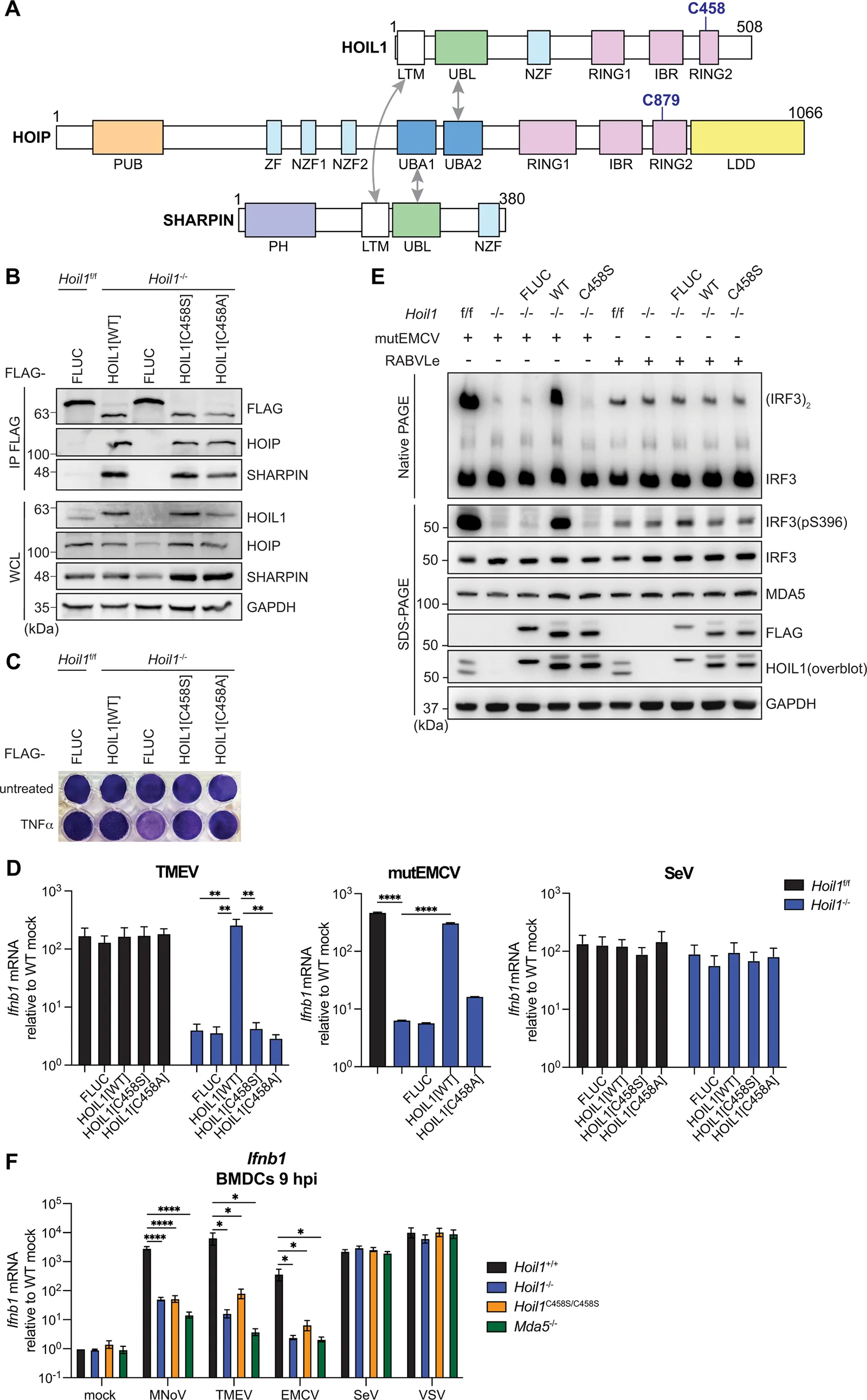
HOIL1 E3 ligase activity is essential for MDA5 signaling and IFN induction. (**A**) Illustration of LUBAC showing domain organization and interactions (to scale). LTM, LUBAC-tethering motif; UBL, ubiquitin-like domain; NZF, Npl4 zinc finger; RING, really interesting new gene; IBR, in-between RING domain; PUB, PNGase/UBA- or UBX-containing domain; UBA, ubiquitin-associated domain; LDD, linear determining domain; PH, pleckstrin homology domain. Arrows indicate domain interactions. Catalytic cysteines for HOIL1 and HOIP are indicated in blue text. (**B**) Immunoblot analyses of LUBAC subunits in anti-FLAG co-immunoprecipitations and whole-cell lysates (WCL) from *Hoil1*^f/f^ and *Hoil1*^−/−^ MEFs stably transduced with vectors expressing 3×FLAG-HOIL1[WT], 3×FLAG-HOIL1[C458S], 3×FLAG-HOIL1[C458A], 3×FLAG-FLUC, or untransduced. (**C**) Crystal violet staining of the indicated MEFs treated with 150 µg/mL TNFα for 48 h, or untreated. (**D**) *Ifnb1* mRNA fold induction in the indicated MEFs after infection with Theiler’s murine encephalomyelitis virus (TMEV) (multiplicity of infection [MOI] of 10; 11 hpi), mutEMCV (MOI of 0.5; 12 hpi), or Sendai virus (SeV) (MOI of 3; 9 hpi), expressed as relative to mock-infected WT cells. (**E**) Native PAGE analysis of IRF3 dimerization (top) and SDS-PAGE analysis of IRF3 phosphorylation, and expression of the indicated proteins (middle and bottom) in the indicated MEFs following infection with mutEMCV (MOI of 5) or transfection with RABV_Le_ RNA (5 pmol/mL) for 8 h. (**F**) *Ifnb1* mRNA fold induction in bone marrow-derived dendritic cells (BMDCs) from WT, *Hoil1*^−/−^, *Hoil1*^C458S/C458S^, or *Mda5*^−/−^ mice infected with murine norovirus (MNoV) (MOI of 3), TMEV (MOI of 3), EMCV strain D (MOI of 3), VSV (MOI of 3), or SeV (MOI of 0.3) for 9 h, relative to mock-infected WT cells. For panels **B, C, and E**, data shown are representative of at least three independent experiments. For panel **D**, TMEV and SeV data are combined means from three to four independent experiments performed in duplicate. mutEMCV data are representative of at least two independent experiments. For panel **F**, data are combined means from four independent experiments performed in duplicate. Means ± SEM are shown. Significance was determined by two-way ANOVA with Tukey’s multiple comparisons test (D [TMEV and SeV]), one-way ANOVA compared to *Hoil1*^−/−^ untransduced with Dunnett’s multiple comparisons test (D [mutEMCV]), or one-way ANOVA with Tukey’s multiple comparisons test (**F**). *, *P* ≤ 0.05; **, *P* ≤ 0.01; ****, *P* ≤ 0.0001.

It has been shown previously that HOIL1 is essential for IFN induction following infection with viruses that activate MDA5 (18). As HOIL1 is essential for the stability and function of its binding partners, HOIP and SHARPIN (23, 26, 48), it remained unclear whether HOIL1 regulates MDA5-dependent IFN induction through its intrinsic E3 ligase activity or indirectly via LUBAC-mediated M1-ubiquitination. To address this, we stably complemented wild-type (*Hoil1*^f/f^) and HOIL1-deficient (*Hoil1*^−/−^) mouse embryonic fibroblasts (MEFs) (49) with FLAG-tagged wild-type HOIL1 (HOIL1[WT]), catalytically inactive mutants (HOIL1[C458S] or HOIL1[C458A]), or firefly luciferase (FLUC) as a control. HOIP and SHARPIN co-immunoprecipitated with FLAG-HOIL1[WT], FLAG-HOIL1[C458S], and FLAG-HOIL1[C458A], confirming that both ligase-dead mutants retained their association with LUBAC (Fig. 1A and B). Because loss of HOIP-mediated M1-ubiquitination sensitizes cells to TNFα-induced cell death (48), we used TNFα sensitivity as a functional readout of LUBAC activity. While *Hoil1*^−/−^ MEFs showed mild TNFα sensitivity due to reduced HOIP expression, expression of HOIL1[WT], HOIL1[C458S], or HOIL1[C458A] restored HOIP and SHARPIN levels and TNFα resistance (Fig. 1B and C). These data confirm that HOIL1 ligase-defective mutants support LUBAC formation and function (40, 41).

To test whether HOIL1 E3 ligase activity is required for MDA5-dependent IFN induction, complemented MEFs were infected with MDA5-activating viruses (TMEV and encephalomyocarditis virus [i.e., mutEMCV, a mutant deficient in MDA5 antagonism]) (50) or a RIG-I-activating virus (SeV), and *Ifnb1* induction was measured. Consistent with previous work (18), *Ifnb1* induction was strongly impaired in *Hoil1*^−/−^ cells following TMEV (2.38% of WT) and mutEMCV (1.37% of WT) infection (Fig. 1D). This defect was rescued by HOIL1[WT] but not by HOIL1[C458S/A], demonstrating a requirement for HOIL1 catalytic activity in MDA5-mediated IFN induction. In contrast, SeV-induced *Ifnb1* expression was not significantly affected (Fig. 1D). Similar results were obtained using transfection of immunostimulatory viral RNAs (51) that predominantly activate MDA5 (TMEV- or SARS-CoV-2-infected cell RNA) or RIG-I (*in vitro*-transcribed rabies virus leader RNA [RABV_Le_] or VSV-infected cell RNA), further supporting a selective requirement for HOIL1 ligase activity in MDA5 signaling (Fig. S1A). Since HOIL1[C458S] and HOIL1[C458A] lack E3 ligase activity (39–41) and behaved similarly in our assays, HOIL1[C458S] was used for subsequent experiments. Consistently, IRF3 phosphorylation and dimerization were markedly reduced in HOIL1[C458S]-reconstituted cells following MDA5 activation, but not following RIG-I activation, indicating that HOIL1 E3 ligase activity functions upstream of IRF3 activation in the MDA5 pathway (Fig. 1E).

Extending these findings to primary immune cells, BMDCs from *Hoil1*^C458S/C458S^ (39), *Hoil1*^−/−^, and *Mda5*^−/−^ mice exhibited strongly reduced *Ifnb1* induction compared to WT BMDCs at 9 h post-infection (hpi) with MNoV, TMEV, or EMCV strain D (EMCV-D), but not after SeV or VSV infection (Fig. 1F). Although subtle differences in *Ifnb1* mRNA induction were observed among *Hoil1*^−/−^, *Hoil1*^C458S/C458S^, and *Mda5*^−/−^ BMDCs following infection with MNoV, TMEV, or EMCV, the overall responses were similarly attenuated, suggesting that HOIL1 ligase activity is a major but potentially not exclusive contributor to MDA5-mediated IFN induction. IFNβ protein levels in BMDC supernatants and induction of ISG and cytokine mRNAs (*Ifit2, Il6*, *Isg15,* and *Cxcl10*) mirrored *Ifnb1* expression, indicating a functional impairment of the antiviral response (Fig. S1B and C). Similar results were also observed at 6 hpi, signifying an early defect (Fig. S1D). Importantly, HOIP and SHARPIN protein levels were comparable between WT and *Hoil1*^C458S/C458S^ cells, confirming that LUBAC was not destabilized (Fig. S1E). Together, these data demonstrate that HOIL1 E3 ubiquitin ligase activity is required for efficient MDA5-dependent antiviral signaling, independent of its role in maintaining LUBAC stability.

### HOIL1 E3 ligase activity is required for the acute IFN response to norovirus and EMCV infection *in vivo*

To determine the relevance of HOIL1 E3 ligase activity in viral infection *in vivo*, we infected 8- to 9-week-old *Hoil1*^+/+^ and *Hoil1*^C458S/C458S^ mice intragastrically with 10^6^ PFU MNoV strain CW3 and examined the IFN response and viral loads. *Mda5*^−/−^ and *Hoil1*^−/−^ mice, and their WT littermates, were included as controls. MNoV CW3 establishes an acute enteric infection, followed by systemic dissemination that is cleared in WT mice (52, 53). At 1 dpi, *Ifnb1* and ISG mRNA induction in the mesenteric lymph nodes (MLN) was significantly lower in *Mda5*^−/−^, *Hoil1*^−/−^, and *Hoil1*^C458S/C458S^ mice relative to WT littermates, while MNoV genome copies were not significantly different (Fig. S2A and B). At 2 dpi, viral genome levels were elevated in MLN from *Mda5*^−/−^ and *Hoil1*^−/−^ mice relative to WT controls (Fig. S2C). However, viral genomes were not elevated in *Hoil1*^C458S/C458S^ MLN relative to controls at 2 or 3 dpi (Fig. S2C and D). Few differences in IFN responses or viral burdens were observed in other tissues, even in *Mda5*^−/−^ mice (not shown).

**Fig 2:**
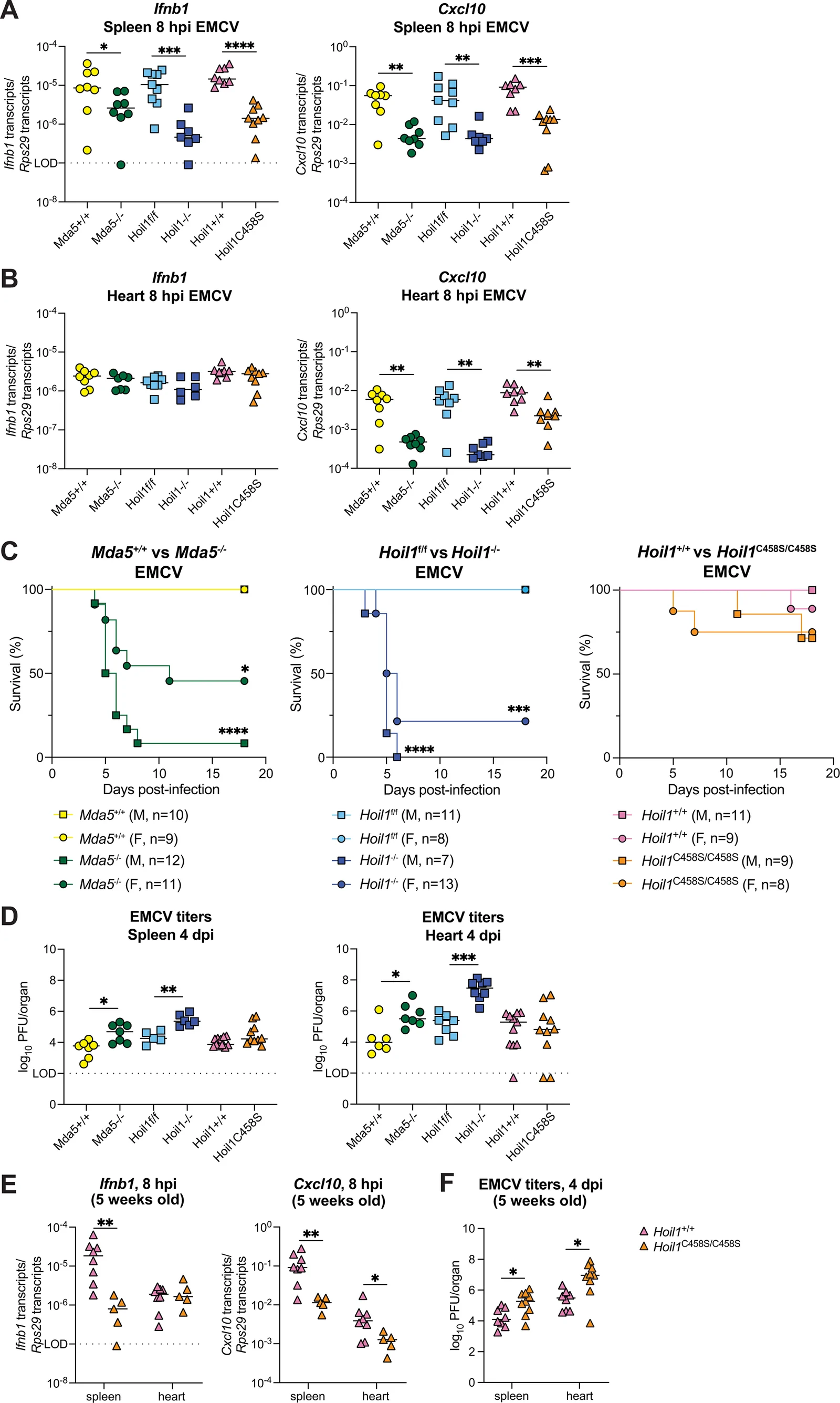
HOIL1 E3 ligase controls the early IFN response during EMCV infection. (**A and B**) *Ifnb1* and *Cxcl10* mRNA at 8 hpi in the spleen (**A**) and heart (**B**) from the indicated 8-week-old mice infected with 10^6^ PFU EMCV-D i.p. (**C**) Percent survival of male (**M**) and female (**F**) 8-week-old *Mda5*^−/−^, *Hoil1*^−/−^, or *Hoil1*^C458S/C458S^ mice and WT littermate controls infected with 5,000 PFU EMCV-D. (**D**) EMCV titers at 4 dpi in the spleen and heart from 8-week-old mice infected with 5,000 PFU. (**E**) *Ifnb1* and *Cxcl10* mRNA at 8 hpi in the spleen and heart of 5-week-old *Hoil1*^C458S/C458S^ and WT control mice infected with 10^6^ PFU EMCV-D i.p. (**F**) EMCV titers at 4 dpi in the spleen and heart from 5-week-old mice infected with 5,000 PFU. LOD, limit of detection. For panels **A, B, D, E,** and **F**, male and female mice were included in all groups. For panels **A**, **B**, **D**, **E**, and **F**, each symbol represents an individual mouse, and the median is indicated. mRNA copies were normalized to *Rps29* mRNA copies. Data are combined from at least three independent experiments. Statistical analyses were performed using Mann-Whitney tests between matched groups (**A, B, D, E**, and** F**) or log-rank (Mantel-Cox) tests (**C**). *, *P* ≤ 0.05; **, *P* ≤ 0.01; ***, *P* ≤ 0.001; ****, *P* ≤ 0.0001.

Surprised by the differences in phenotypes during MNoV infection, we next examined responses to infection with EMCV-D, which has low virulence in WT C57BL/6 mice but causes severe disease in MDA5-deficient mice (54). Following intraperitoneal infection of 8-week-old mice with 10^6^ PFU, induction of *Ifnb1* and *Cxcl10* in the spleen, and *Cxcl10* in the heart, was significantly lower in *Mda5*^−/−^, *Hoil1*^−/−^, and *Hoil1*^C458S/C458S^ mice at 8 hpi relative to WT littermates (Fig. 2A and B; Fig. S3A and B), demonstrating that HOIL1 ligase activity is required for an optimal early IFN response *in vivo*. Despite this defect, *Hoil1*^C458S/C458S^ mice exhibited minimal weight loss and morbidity following infection with 5,000 PFU EMCV-D, whereas *Mda5*^−/−^ and *Hoil1*^−/−^ mice developed severe disease beginning around 4 dpi (Fig. 2C; Fig. S3C). At 4 dpi, EMCV titers were significantly higher in the spleen and heart of *Mda5*^−/−^ and *Hoil1*^−/−^ mice relative to WT controls, but only modestly increased in the spleens of *Hoil1*^C458S/C458S^ mice (Fig. 2D). Because younger mice are more susceptible to EMCV-D infection, we also infected 5-week-old *Hoil1*^C458S/C458S^ mice. Early IFN and ISG responses were impaired, and EMCV titers in the spleen and heart were significantly increased (Fig. 2E and F), although weight loss and survival were similar to those of WT mice (Fig. S3D and E). Together, these findings demonstrate that HOIL1 ligase activity is required for efficient IFN responses during MNoV and EMCV infection and contributes to control of EMCV replication, particularly in juvenile mice. We speculate that phenotypic differences between *Hoil1*^C458S/C458S^ and *Hoil1*^−/−^ mice, despite similarly reduced IFN responses, may be due to differences in expression or activity of HOIP (Fig. S1E), which regulates multiple signaling pathways that likely influence viral infection (19, 20, 23, 25, 27, 30, 39, 40, 46), or that HOIL1 has additional functions yet to be determined.

### HOIP plays a minor role in RLR antiviral signaling

To determine whether HOIP E3 ligase activity influences MDA5 signaling, we generated *Hoip*^−/−^ MEFs using CRISPR-Cas9-mediated disruption of exon 3, encoding part of the N-terminal PUB domain. As expected, *Hoip*^−/−^ MEFs were more sensitive to TNFα-induced cell death than *Hoil1*^−/−^ cells (Fig. S4A) (29, 55). Following TMEV-RNA transfection, *Ifnb1* mRNA induction in *Hoip*^−/−^ MEFs was reduced to ~20% of WT cells, compared to a <1% reduction in *Hoil1*^−/−^ cells (Fig. S4B), indicating that HOIP plays a less prominent role than HOIL1 in MDA5 signaling.

To assess the requirement for HOIP catalytic activity, *Hoip*^−/−^ MEFs were complemented with FLAG-tagged HOIP[WT], ligase-dead HOIP[C879S], or HOIP[C879A] (36, 37, 56), or FLUC. HOIL1 and SHARPIN co-immunoprecipitated with HOIP[C879S] and HOIP[C879A], similar to HOIP[WT], demonstrating that HOIP catalytic mutants were incorporated into the LUBAC complex (Fig. S4C). Whereas HOIP[WT] restored resistance to TNFα-induced cell death in *Hoip*^−/−^ cells, neither ligase-dead mutant did so, confirming that either mutation disrupts HOIP E3 ligase activity (Fig. S4D) (29). We next examined RLR signaling in these cells. HOIP[WT], but not HOIP[C879S] or FLUC, increased *Ifnb1* induction in response to both TMEV RNA and VSV RNA transfection (Fig. S4E and F), indicating that, unlike HOIL1, HOIP ligase activity contributes modestly to both MDA5- and RIG-I-dependent IFN induction, consistent with recent reports (35, 57).

### HOIL1 can regulate MDA5 signaling independent of its association with HOIP

**Fig 3:**
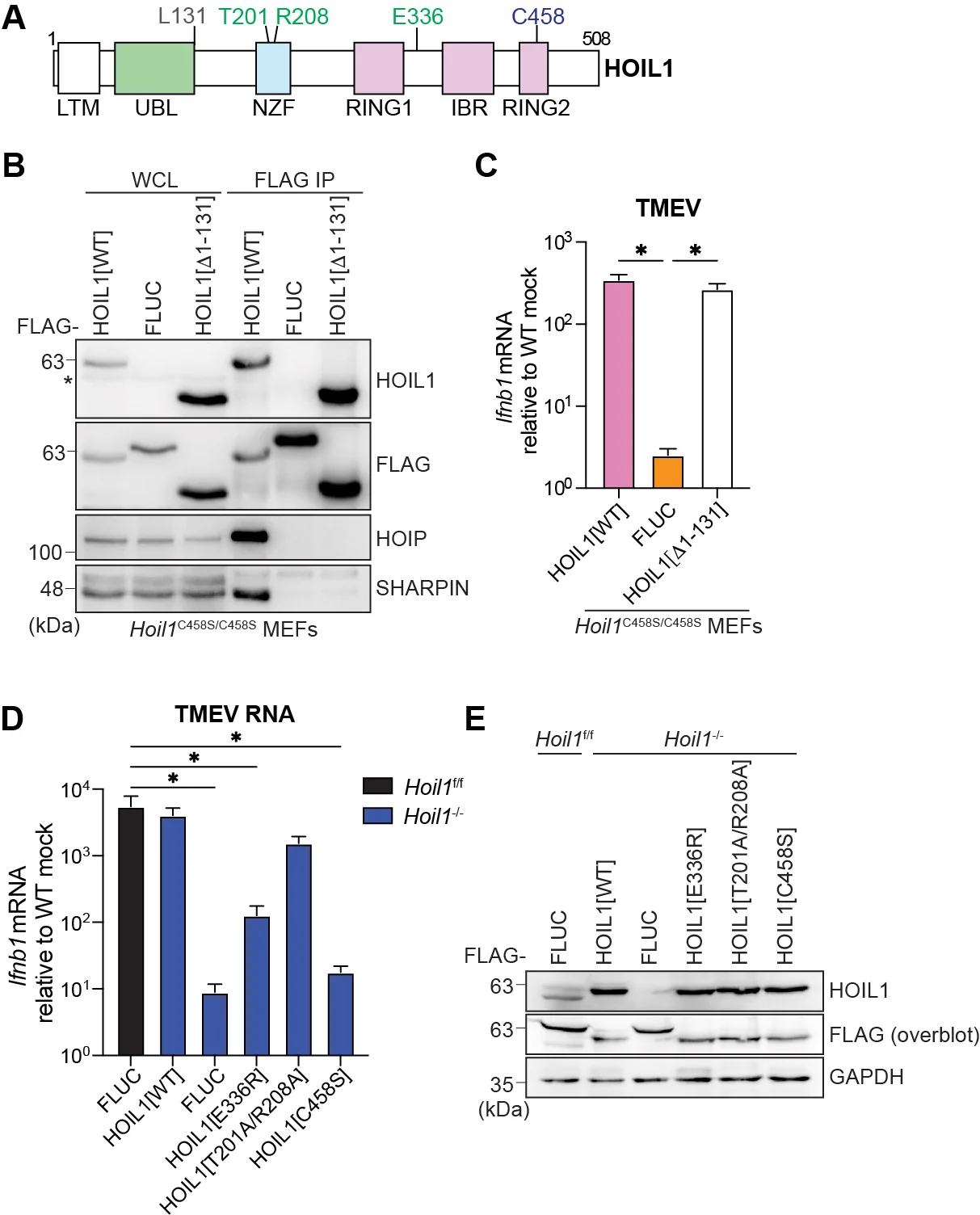
HOIL1 interaction with HOIP is dispensable for facilitation of MDA5 signaling. (**A**) Illustration of HOIL1 domain organization. The locations of the end of the UBL domain (L131, gray), the catalytic cysteine (blue), and key residues involved in ubiquitin binding (green) are indicated. (**B and C**) *Hoil1*^C458S/C458S^ MEFs were transduced with 3×FLAG-HOIL1[WT], 3×FLAG-HOIL1[Δ1–131], or 3×FLAG-FLUC. LUBAC subunit expression was analyzed in whole-cell lysates and after FLAG-IP (**B**). *, endogenous HOIL1[C458S]. *Ifnb1* induction was measured at 15 h post-TMEV infection (MOI of 3) (**C**). (**D and E**) *Hoil1*^−/−^ MEFs were transduced with 3×FLAG-FLUC, 3×FLAG-HOIL1[WT], or the indicated HOIL1 mutants. *Hoil1*^f/f^ cells expressing 3×FLAG-FLUC were included as a control. *Ifnb1* mRNA induction was measured at 8 h post-TMEV RNA transfection (**D**). Immunoblot analysis of expression of the indicated HOIL1 variants and FLUC in uninfected MEFs (**E**). For panels **B** and **E**, data shown are representative of three independent experiments. For panels **C and D**, data are combined from three to five independent experiments performed in duplicate, and means ± SEM are shown. Significance was determined by one-way ANOVA with Tukey’s multiple comparisons test. *, *P* ≤ 0.05; **, *P* ≤ 0.01.

HOIL1 deficiency caused a substantially greater defect in MDA5-dependent IFN induction than HOIP deficiency, suggesting that HOIL1 may function independently of LUBAC. Indeed, a fraction of cellular HOIL1 exists outside of LUBAC (40). To test whether association with HOIP is required for MDA5 signaling, we expressed FLAG-tagged HOIL1 lacking the N-terminal “LTM” and UBL domains required for interaction with SHARPIN and HOIP (HOIL1[Δ1–131]) in *Hoil1*^C458S/C458S^ MEFs (Fig. 3A and B) (58–60). HOIL1[WT] and FLUC were included as positive and negative controls, respectively. Immunoblot analysis showed that HOIP and SHARPIN were expressed similarly in all three cell lines (Fig. 3B), likely due to their association with endogenous HOIL1[C458S]. Co-immunoprecipitation confirmed that while HOIL1[WT] interacted with both LUBAC components, HOIL1[Δ1–131], retaining the catalytic site, did not (Fig. 3B). HOIL1[Δ1–131] restored *Ifnb1* induction following TMEV infection as efficiently as HOIL1[WT], demonstrating that HOIL1 can promote MDA5 signaling independent of its association with LUBAC (Fig. 3C).

Because HOIL1 ubiquitin ligase activity can be allosterically activated by binding of M1-linked polyubiquitin (42, 44, 45, 61), yet HOIP ligase activity contributed only modestly to IFN induction, we next examined whether ubiquitin binding by HOIL1 is required for MDA5 signaling. HOIL1 contains two ubiquitin-binding regions: the NZF domain, which preferentially recognizes M1-linked polyubiquitin, and the RING1-IBR region and intervening linker region, which binds both M1- and K63-linked ubiquitin chains (42, 44, 45). Disruption of ubiquitin binding by the NZF domain (HOIL1[T201A/R208A]) (45, 61) had little effect on *Ifnb1* induction following TMEV-RNA stimulation (Fig. 3D and E). In contrast, HOIL1[E336R], which contains a mutation within the ubiquitin-binding interface in the RING1-IBR linker (45), only partially restored *Ifnb1* induction (Fig. 3D and E), indicating that ubiquitin binding by the RING1-IBR region, but not the NZF domain, is important for HOIL1-mediated MDA5 signaling.

### HOIL1 E3 ligase activity is necessary for MDA5-mediated MAVS activation

**Fig 4:**
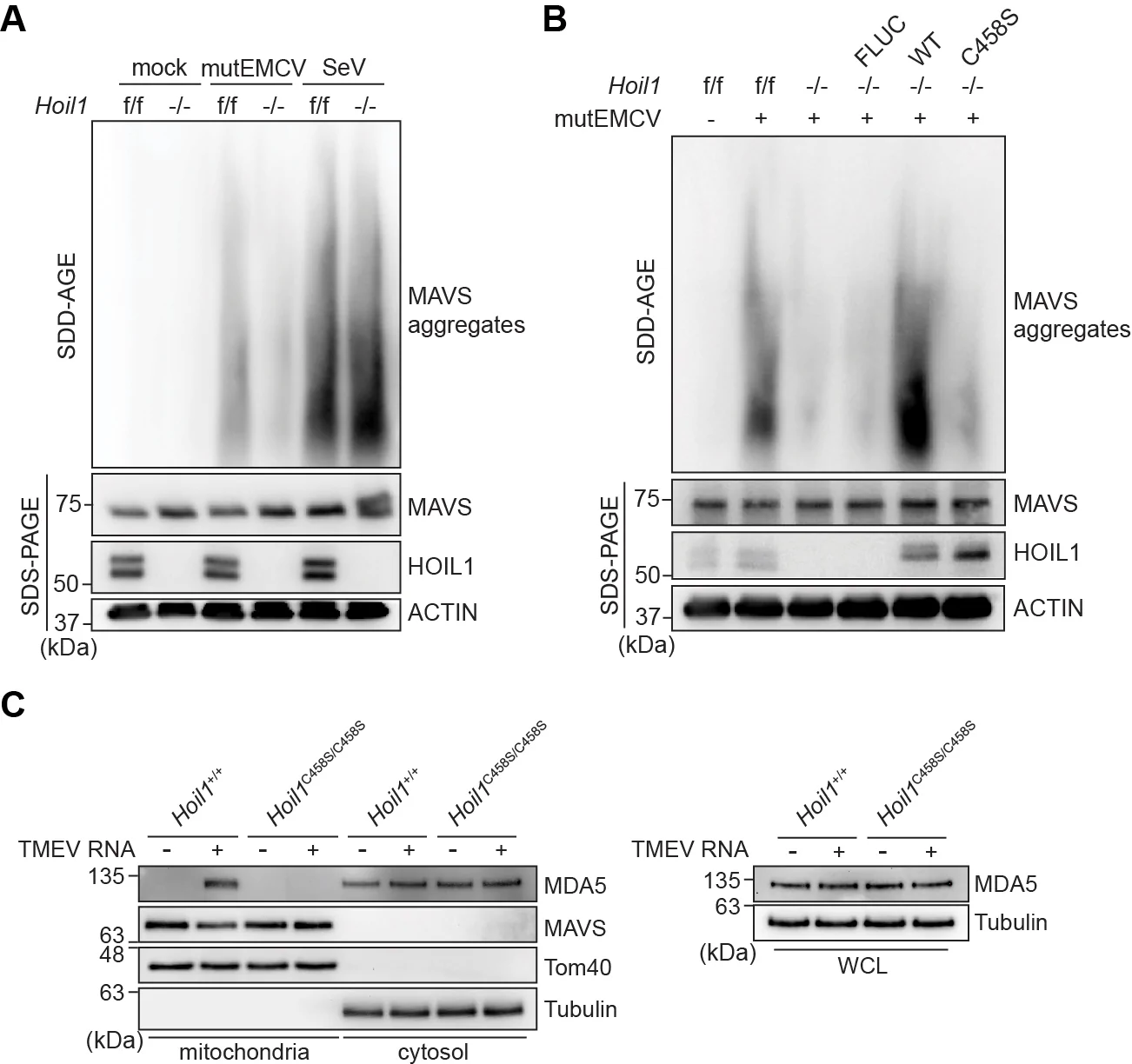
HOIL1 E3 ligase activity promotes MDA5 translocation to mitochondria and activation of MAVS. (**A**) SDD-AGE (top) and SDS-PAGE (bottom) followed by immunoblot analyses of MAVS aggregation and total protein expression in *Hoil1*^f/f^ and *Hoil1*^−/−^ MEFs infected with mutEMCV (MOI of 0.5) or SeV (10 HAU/mL), or mock infected, for 12 h. (**B**) *Hoil1*^f/f^ MEFs, or *Hoil1*^−/−^ MEFs complemented with FLUC, HOIL1[WT], or HOIL1[C458S], were infected with mutEMCV (MOI of 0.5, 12 hpi), and MAVS aggregation was examined as in panel **A**. Uninfected control was included. (**C**) Immunoblot analysis of the indicated proteins in mitochondrial and cytosolic fractions (left) and whole-cell lysates (right) from *Hoil1*^+/+^ (WT) and *Hoil1*^C458S/C458S^ MEFs mock-transfected or transfected with TMEV RNA (4 h). Data shown are representative of two to three independent experiments.

To determine which step in the MDA5 signaling pathway requires HOIL1 E3 ligase activity, we next assessed its effect on MDA5-mediated activation of MAVS using semi-denaturing detergent agarose gel electrophoresis (SDD-AGE) assays to detect MAVS aggregates. Indeed, MAVS aggregation was impaired in *Hoil1*^−/−^ MEFs following mutEMCV, but not SeV, infection (Fig. 4A). Reconstitution with HOIL1[WT], but not HOIL1[C458S], restored MAVS aggregation in response to mutEMCV infection (Fig. 4B). Because MAVS is tethered to the outer mitochondrial membrane, we examined whether HOIL1 ligase activity promotes MDA5 recruitment to mitochondria. Endogenous MDA5 was enriched in the mitochondrial fraction from WT MEFs, but not from *Hoil1*^C458S/C458S^ MEFs, following TMEV-RNA transfection (Fig. 4C). These results indicate that HOIL1 E3 ligase activity promotes MDA5 recruitment to mitochondria and subsequent MAVS activation.

### HOIL1 promotes ubiquitination of LGP2

**Fig 5:**
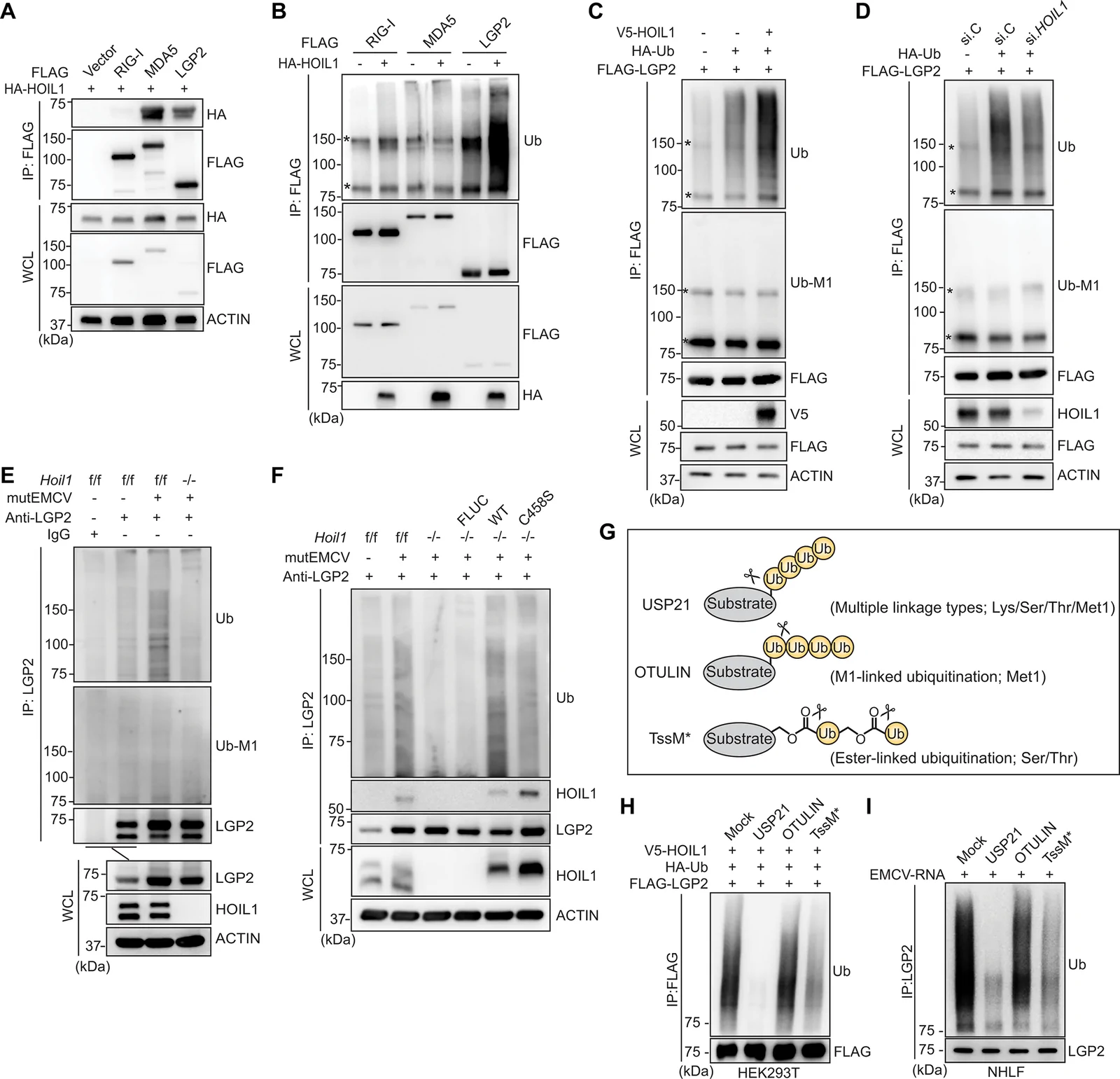
HOIL1 mediates ester-linked ubiquitination of LGP2. (**A**) Co-immunoprecipitation of HA-HOIL1 with FLAG-tagged RLRs (IP: FLAG). Protein expression in WCL is shown below. (**B**) Ubiquitination of FLAG-tagged RLRs immunoprecipitated from 293T cells (IP: FLAG) with or without overexpression of HA-HOIL1. Amounts of immunoprecipitated proteins, or protein expressions or in WCL, are also shown. (**C and D**) Total ubiquitination (top) and M1-ubiquitination (Ub-M1, middle) of immunoprecipitated FLAG-LGP2 in the presence or absence of exogenous HA-ubiquitin, either after co-expression of V5-HOIL1 (**C**) or after KD of *HOIL1* (**D**) in 293T cells. Protein expression in WCL is shown below. (**E**) Ubiquitination of endogenous LGP2 immunoprecipitated from WT (f/f) and *Hoil1*^−/−^ MEFs with and without mutEMCV infection (MOI of 0.1, 12 hpi). Total ubiquitination (top) and M1-ubiquitination (middle) are shown. Protein expression in WCL is shown below. (**F**) Ubiquitination of endogenous LGP2 immunoprecipitated from WT (f/f) or *Hoil1*^−/−^ MEFs complemented with FLUC, HOIL1[WT] or HOIL1[C458S], with and without mutEMCV infection (MOI of 0.1, 12 hpi). (**G**) Schematic depicting cleavage of ubiquitin linkage types by deubiquitinases (DUBs) USP21, OTULIN and TssM*. (**H and I**) Deubiquitination of LGP2 by USP21, OTULIN, or TssM*. LGP2 was immunoprecipitated from 293T cells transfected for 24 h with FLAG-LGP2, V5-HOIL1, and HA-Ub (**H**), or from normal human lung fibroblasts (NHLFs) transfected with EMCV-RNA (400 ng/mL) for 16 h (**I**) and then incubated with the indicated DUBs (1 μM) for 30 min. Ubiquitination of affinity-purified LGP2 was determined by anti-Ub immunoblot. Asterisks in panels **B–D** indicate non-specific bands. Data shown are representative of at least two independent experiments.

Our findings indicated that HOIL1 E3 ligase activity regulates an early step in MDA5 activation. We therefore investigated whether HOIL1 directly modifies MDA5 or a key regulator of its activation. siRNA-mediated knockdown (KD) of endogenous *HOIL1* in 293T cells did not affect MDA5 ubiquitination or ISGylation following stimulation with high molecular weight poly(I:C), which primarily activates MDA5 (Fig. S5A and B). Given the essential role of LGP2 in promoting MDA5 signaling (10, 13, 14), we next examined whether HOIL1 targets LGP2. HA-HOIL1 co-immunoprecipitated with both FLAG-MDA5 and FLAG-LGP2, but not FLAG-RIG-I (Fig. 5A). To test whether HOIL1 can ubiquitinate LGP2, FLAG-RLRs were expressed in the presence or absence of ectopic HOIL1, followed by immunoprecipitation and analysis of ubiquitination. Ectopic HOIL1 expression enhanced the ubiquitination of LGP2 (Fig. 5B and C) but not MDA5 or RIG-I (Fig. 5B). Inversely, KD of endogenous *HOIL1* reduced ubiquitination of FLAG-LGP2 (Fig. 5D). Similarly, mutEMCV infection induced HOIL1-dependent ubiquitination of endogenous LGP2 in MEFs (Fig. 5E). Notably, we did not detect M1-polyubiquitination of LGP2, consistent with our findings that HOIL1 functions independently of LUBAC (Fig. 5C through E). Furthermore, reconstitution of HOIL1-deficient MEFs with HOIL1[WT], but not HOIL1[C458S], restored LGP2 ubiquitination during mutEMCV infection (Fig. 5F), demonstrating that HOIL1 E3 ligase activity is required for LGP2 ubiquitination.

To determine whether HOIL1 catalyzes ester-linked ubiquitination of LGP2, we treated ubiquitinated LGP2 with DUBs targeting defined ubiquitin-linkage types (Fig. 5G). TssM*, a V466R mutant of the *Burkholderia* bacterial DUB TssM, selectively cleaves ester-linked ubiquitin modifications with minimal activity toward lysine-linked modifications (62), whereas USP21 cleaves multiple ubiquitin-linkage types and OTULIN specifically hydrolyzes M1-linked polyubiquitin (63, 64). USP21 removed nearly all polyubiquitin associated with FLAG-LGP2 affinity purified from cells expressing exogenous HOIL1 and HA-ubiquitin, as expected (Fig. 5H). TssM* removed most, but not all, polyubiquitin from LGP2, whereas OTULIN had little effect. Similar results were observed for endogenous LGP2 isolated from EMCV-RNA-transfected NHLFs (Fig. 5I). Together, these data identify LGP2 as a substrate for HOIL1-mediated ester-linked ubiquitination.

To corroborate that HOIL1 promotes MDA5 activation through LGP2, we knocked down endogenous *Lgp2* in *Hoil1*^−/−^ MEFs expressing HOIL1[WT], HOIL1[C458S], or FLUC (control), and assessed MDA5 oligomerization and MAVS aggregation following EMCV-RNA transfection. HOIL1[WT], but not HOIL1[C458S], promoted the formation of MDA5 oligomers and MAVS aggregates in cells transfected with control siRNA (Fig. S5C and D). Knockdown of *Lgp2* almost completely abolished both MDA5 oligomerization and MAVS aggregation in all of the cell lines, consistent with previous studies (Fig. S5C and D) (13, 14). Consistently, *Lgp2* silencing diminished *Ifnb1*, *Ifna2,* and *Isg15* mRNA induction in cells expressing HOIL1[WT], but had little additional effect in cells expressing HOIL1[C458S] (Fig. S5E and F). These findings indicate that HOIL1 ligase activity and LGP2 function at a similar step of the signaling pathway to promote MDA5 oligomerization, MAVS activation, and antiviral gene expression.

Collectively, our findings support a model in which HOIL1 regulates MDA5 signaling by promoting ester-linked ubiquitination of LGP2 (Fig. S5G). Future studies will need to test if and how ubiquitination of a specific residue(s) in LGP2 regulates MDA5 activation.

## DISCUSSION

MDA5 signaling is essential for the induction of protective antiviral responses to a range of RNA viruses. However, aberrant MDA5 activation can drive inflammation in patients with Singleton-Merten syndrome and Aicardi-Goutières syndrome and is implicated in the pathogenesis of other immune disorders, such as systemic lupus erythematosus, ADAR1 deficiency, and type 1 diabetes (15, 16, 65, 66). A detailed understanding of the mechanisms controlling MDA5 signaling is therefore important for developing strategies to enhance antiviral immunity and to limit auto-inflammation.

In this study, we define the role of HOIL1 in MDA5 antiviral signaling by delineating the relative contributions of HOIL1 E3 ubiquitin ligase activity and LUBAC (HOIP) activity. Previous studies on LUBAC in RIG-I signaling have reported conflicting results, likely reflecting differences in experimental systems, cell types or receptor agonists, and incomplete deletion of LUBAC components (18, 31–35). Consistent with our findings, several studies have described partial defects in RIG-I-mediated IFN induction in the absence of HOIP or its catalytic activity (34, 35). We extend these observations by showing that HOIP ligase activity also contributes to MDA5 signaling, suggesting a shared requirement for HOIP-mediated M1-polyubiquitin downstream of both RLRs. This is consistent with models in which HOIP-generated M1-linked polyubiquitin promotes recruitment of NEMO to MAVS, enabling activation of IRF3 and NF-κB (34, 35), and with reports that HOIP ubiquitinates TRAF6 downstream of MAVS (57).

HOIL1, in contrast, catalyzes non-canonical ester-linked ubiquitination, yet its substrates and physiological roles are poorly defined (38–43, 47, 67, 68). Here, we demonstrate that HOIL1 ubiquitin ligase activity is required for MDA5-dependent antiviral responses induced by multiple viruses. HOIL1 can exist independently of LUBAC (69, 70), and our data indicate a LUBAC-independent role in antiviral signaling. Consistent with *in vitro* evidence that M1- or K63-polyubiquitin binding promotes HOIL1 ligase activity (44, 45), we find that the polyubiquitin binding region between the HOIL1 RING1 and IBR domains is important for IFN induction. Notably, HOIP E3 ligase activity was not absolutely required for this function, implying that other E3 ligase(s) may generate polyubiquitin scaffolds that enable HOIL1 activity.

Mechanistically, HOIL1 acts upstream of MAVS and promotes MDA5 oligomerization during infection (Fig. S5G). We identify LGP2, a key positive regulator of MDA5 filament assembly (13, 14), as a substrate for ester-linked ubiquitination by HOIL1. Although HOIL1-dependent ubiquitination of LGP2 is robustly detected, residual modification in the presence of the HOIL1 catalytic mutant and incomplete removal by an ester-specific deubiquitinase suggest that additional enzymes or linkages may contribute. We did not detect M1-linked ubiquitination of LGP2, and OTULIN had no effect, supporting a mechanism independent of HOIP-generated M1-polyubiquitin chains.

Our findings align with the established role of LGP2 in promoting MDA5 RNA binding and filament nucleation (13, 14). LGP2 binds dsRNA with higher affinity than MDA5 (9, 13), and can facilitate MDA5 filament assembly and conformational activation (14). In addition, LGP2 negatively regulates RLR signaling later in infection by inhibiting TRAF ubiquitin ligases downstream of MAVS (11, 12). Our data place HOIL1 E3 ligase activity upstream of MAVS and indicate that it does not affect RIG-I signaling. However, the precise site(s) and consequences of LGP2 ubiquitination remain unknown, and future studies will also be required to determine whether HOIL1 directly modifies LGP2 or acts on pre-conjugated ubiquitin chains, as well as how this modification influences MDA5 RNA binding and signaling.

*In vivo*, HOIL1 plays a more complex role in antiviral immunity. HOIL1 ligase activity is required for optimal type I IFN responses during MNoV and EMCV infection of mice, similar to MDA5. However, phenotypic differences between *Hoil1*^−/−^ mice (lacking the HOIL1 NZF and RING-BETWEEN-RING domains and expressing reduced HOIP and SHARPIN) and *Hoil1*^C458S/C458S^ mice suggest additional functions. In *Hoil1*^−/−^ mice, the associated reduction in HOIP and SHARPIN expression likely impairs multiple innate immune and inflammatory pathways, thereby increasing susceptibility to infection-associated pathology (19, 20, 23, 25–27, 29, 30). In contrast, mice lacking only HOIL1 ligase activity exhibit enhanced HOIP ligase activity and M1-ubiquitination (39, 40, 46, 47), which has been associated with increased inflammation, particularly in older mice, and reduced apoptotic cell death (40, 46). Consistent with this, we observed elevated inflammatory expression in the heart of uninfected *Hoil1*^C458S/C458S^ mice and age-dependent differences in viral susceptibility. We speculate that the altered balance of M1-polyubiquitin in these mice may influence cell death pathways, particularly in the heart where EMCV induces pathology in MDA5-deficient mice (54). Additional contributions from genetic background and microbiome composition may further influence these phenotypes.

Together, our study identifies HOIL1 as a key regulator of MDA5-dependent antiviral signaling and establishes LGP2 as a substrate for HOIL1-mediated ester-linked ubiquitination, revealing a new layer of regulation within the RLR pathway.

## MATERIALS AND METHODS

### Mice

*Hoil1*^f/f^ and *Hoil1*^−/−^ mice were derived from B6NCrl; B6N-A^tm1Brd^*Rbck1^tm1a(EUCOMM)Hmgu^* mice and have been described previously (49). *Mda5*^−/−^ (B6.Cg-*Ifih1^tm1.1Cln^*/J, stock #015812) mice were purchased from Jackson Laboratories (Bar Harbor, ME, USA). *Hoil1*^C458S/C458S^ KI mice were a gift from Philip Cohen (39).

### Viral strains

Stocks of MNoV strain CR6 and CW3 were generated from molecular clones as previously described (71, 72) and amplified twice in BV2 cells. Sendai virus Cantell strain was obtained from ATCC (VR-907). VSV-GFP (73) and TMEV strain GDVII were propagated in Vero cells and BHK-21 cells, respectively. EMCV-D was a gift from John Corbett and propagated in L929 cells. mutEMCV, which carries two-point mutations (C19A/C22A) in the zinc domain of the L protein, was kindly provided by F. J. M. van Kuppeveld (Utrecht University) and was propagated in BHK-21 cells (50). Viral titers were determined by plaque assay. TMEV and VSV RNAs were isolated from virally infected BHK-21 cells using TRI-reagent. EMCV RNA, SARS-CoV-2 RNA, and RABV_Le_ RNA were generated as previously described (74–76).

### Plasmids and cloning

*Hoip* (*Rnf31*) cDNA was PCR amplified from mouse cDNA, and FLUC was amplified from pTrip-CMV-FLUC-EYFP (77). 3×FLAG-*Hoil1* (*Rbck1*) cDNA was synthesized as a gene block (IDT). *Hoil1* and *Hoip* mutations and deletions were generated using the Q5 site-directed mutagenesis kit (New England Biolabs). FLAG-tagged cDNAs were transferred into pTrip-CMV-Puro-T2A-RFP (77) or pSCRBBL (derivative of pSCRPSY carrying the blasticidin resistance gene) lentiviral vectors using Gateway cloning (Invitrogen). FLAG-hMDA5, FLAG-hRIG-I, HA-Ub, pCAGGS–HA–Ube1L, pFLAG–CMV2–UbcH8, and V5-hISG15 have been described previously (75, 76). Human LGP2 was amplified from cDNA and cloned into pcDNA3.1-FLAG. HA-hHOIL1 and V5-hHOIL1 were kindly provided by J. U. Jung (Cleveland Clinic) and have been described previously (32).

### Cell culture, treatments, and infections

MEFs, BHK-21 cells, Vero cells, L929 cells, HEK293T cells, and NHLFs were cultured in DMEM supplemented with 10% FBS, 1% L-glutamine, and 0.5% penicillin/streptomycin at 37°C with 5% CO_2_.

Transient gene knockdown in HEK293T and MEFs was performed using SMARTpool small interfering RNAs (siRNAs) (Horizon Discovery) as previously described (78). These include specific siRNAs targeting human *HOIL1* (L-006932-00-0005), mouse *Lgp2* (L-050413-00-0005), and non-targeting control pool (D-001810-10-05). For transient overexpression in HOIL1-knockdown HEK293T cells, cells were first transfected with HOIL1-targeting siRNAs for 48 h, followed by transfection with the indicated plasmids for an additional 24 h. Poly(I:C) was transfected 6 h before cell harvest.

For viral infections, MEFs were plated in tissue culture-treated plates and infected 20 h later with SeV (MOI of 3), VSV-GFP (MOI of 3), TMEV GDVII (MOI of 3 or 10), or mutEMCV (MOI of 0.5). Alternatively, TMEV and VSV RNA isolated from infected BHK-21 cells was transfected into cells at 50 ng/mL using the *Trans*IT-mRNA transfection kit (Mirus). RABV_Le_ RNA and EMCV RNA were transfected with Lipofectamine RNAiMAX Transfection Reagent (Invitrogen) as previously described (75).

BMDCs were differentiated in RPMI-1640 (Corning) supplemented with 10% FBS, 1% L-glutamine, and 2% conditioned media from J558L cells containing GM-CSF in a 37°C, 5% CO_2_, humidified incubator for 7 days. Non-adherent cells were collected and infected with MNoV CR6, TMEV GDVII, EMCV-D, or VSV-GFP at an MOI of 3, with SeV at an MOI of 0.3 (0.3 EID_50_/cell), or mock-infected, and re-plated in tissue culture-treated dishes.

At the indicated time points, cells were lysed in TRI-reagent (Millipore-Sigma) for RNA extraction, or in 2× Laemmli buffer then boiled for 10 min for immunoblot analyses. Cell culture supernatants were stored at −80°C until analysis.

### RNA isolation and quantitative reverse transcription-PCR

Cells were lysed in TRI-reagent, or frozen tissues were homogenized in 1 mL TRI-reagent, and RNA was isolated according to the manufacturer’s instructions. RNA samples were treated with Turbo DNA-free DNase (Invitrogen) and used as a template for cDNA synthesis with ImProm-II reverse transcriptase (Promega). qPCR was performed using a QuantStudio 3 Real-Time PCR System using Amplitaq Gold (Applied Biosystems) and predesigned probe-based assays (IDT, Table 1). *Rps29* and MNoV TaqMan qPCR assays were described previously (18, 79). MNoV genome copies and mRNA levels were determined relative to standard curves and normalized to *Rps29,* or by ΔΔ*C*_*T*_ relative to mock.

**TABLE 1:** Probe-based qPCR assays (IDT)

Gene	Assay ID
*Ifnb1*	Mm.PT.58.30132453.g
*Ifit2*	Mm.PT.58.28800045.g
*Il6*	Mm.PT.58.10005566
*Cxcl10*	Mm.PT.58.43575827
*Ifng*	Mm.PT.58.41769240
*Tnf*	Mm.PT.58.12575861
*Ccl2*	Mm.PT.58.42151692
*Isg15*	Mm.PT.58.41476392.g
*Ifna2*	Mm.PT.58.45839156.g

### Co-immunoprecipitations

For IP of proteins in transiently transfected HEK293T cells and of LUBAC in MEFs, cells were lysed in NP40 buffer (1% [vol/vol] NP-40, 150 mM NaCl, 50 mM Tris-HCl (pH 7.5), 1 mM EDTA). Lysates were centrifuged at 10,000 × *g* for 12 min, and the supernatant was incubated with anti-FLAG magnetic agarose and further processed according to the manufacturer’s instructions (Pierce A36797). For endogenous LGP2 IP, MEFs were infected with mutEMCV (MOI of 0.1) for 12 h, and cell lysates were prepared and precleared with Protein G Dynabeads at 4°C for 2 h. The lysates were incubated with Protein G Dynabeads pre-conjugated with anti-LGP2 (Proteintech, 11355-1-AP) or normal rabbit IgG (CST, 2729S) at 4°C for 4 h. Beads were washed four times with RIPA buffer (20 mM Tris-HCl, pH 8.0, 150 mM NaCl, 1% [vol/vol] NP-40, 1% [wt/vol] deoxycholic acid, and 0.01% SDS), and proteins were eluted in 1× Laemmli SDS sample buffer. Protein samples were separated by SDS-PAGE, transferred onto PVDF membranes, and analyzed by immunoblotting.

### Mitochondrial fractionation

Mitochondrial fractionation was performed using a mitochondria isolation kit (Thermo Scientific) according to the manufacturer’s instructions, with minor modifications.

### SDD-AGE

MAVS aggregation in MEFs was assessed by SDD-AGE as previously described (75, 80).

### IRF3 dimerization

Endogenous IRF3 dimerization was determined by native PAGE as previously described (81).

### Deubiquitinase assays

HEK293T cells were co-transfected with FLAG-LGP2, V5-HOIL1, and HA-ubiquitin for 24 h. FLAG-tagged LGP2 was immunoprecipitated and immobilized using anti-FLAG beads as described above. Equal amounts of immobilized FLAG-LGP2 were incubated with USP21, OTULIN, or TssM* (TssM V466R mutant) (1 μM each) at 37°C for 30 min followed by immunoblot. NHLFs were transfected with EMCV-RNA (400 ng/mL) for 16 h. Endogenous LGP2 was immunoprecipitated and immobilized using anti-LGP2 antibody-coated resin as described above. Equal amounts of immobilized LGP2 were then incubated with USP21, OTULIN, or TssM* (1 μM each) at 37°C for 30 min and analyzed by immunoblot. Recombinant USP21, OTULIN, and TssM* were expressed, purified, and activated with reducing agent before use as previously described (62–64).

### Infection of mice

Mice (8–9 weeks old) were inoculated i.g. with 10^6^ PFU of MNoV CW3 in 100 μL of DMEM. Mice (8–9 weeks old or 5 weeks old) were inoculated i.p. with 10^6^ or 5,000 PFU EMCV-D in 300 μL DMEM. For weight-loss and morbidity studies, mice were weighed at the time of infection and each subsequent day. Mice that had lost more than 20% of their initial weight or became moribund were humanely euthanized. Tissues from euthanized mice were harvested into sterile O-ringed tubes containing 1 mm zirconia/silica beads, then flash-frozen in a bath of ethanol and dry ice and stored at −80°C. Viral titers were determined by plaque assay.

### Statistical analyses

Data were analyzed with Prism 10 software (GraphPad Software, San Diego, CA, USA) as described in the legends for Fig. 1–3 and Fig. S1–S5.

Additional method details are included in the Supplemental methods.

## Data Availability

All data used in this study are included in the article and supplemental material.

## SUPPLEMENTAL MATERIAL

Supplemental material mbio.02152-26-s0001.pdf Supplemental methods and Figures S1 to S5.

ASM does not own the copyrights to Supplemental Material that may be linked to, or accessed through, an article. The authors have granted ASM a non-exclusive, world-wide license to publish the Supplemental Material files. Please contact the corresponding author directly for reuse.
