# Supplementary material for: Atypical E3 ligase HOIL1 promotes MDA5 signaling and ester-linked ubiquitination of LGP2
Source: mBio. 2026 Oct 22;17(11):e02152-26. doi: 10.1128/mbio.02152-26 (PMC13753823; doi:10.1128/mbio.02152-26)
Supplement: Supplementary file 1 [file mbio.02152-26-s0001.pdf]

Supplementary Material For:

Atypical E3 ligase HOIL1 promotes MDA5 signaling and ester-linked ubiquitination of LGP2

Deion Cheng, Junji Zhu, W. A. Gayan Chathuranga, Surajit Chakraborty, GuanQun Liu, Rachel E. Lacoursiere, Jonathan N. Pruneda, Michaela U. Gack, Donna A. MacDuff

Supplementary Methods and Materials

Supplementary References

Supplementary Figures 1-5

Supplementary Methods and Materials

Plasmids and cloning. *Hoip* (*Rnf31*) and Fluc cDNA PCR products were cloned into p3xFLAG-CMV-10 (Sigma) NotI/XbaI. FLAG-tagged DNA constructs were PCR amplified and cloned into pDONR221 using BP Clonase (Invitrogen), prior to transfer into lentiviral vectors. LentiCRISPR v2 [a gift from Feng Zhang (Addgene plasmid # 52961) (1)] expressing gRNA targeting *Hoip* exon 3 was created using complementary DNA oligos (IDT) with the sequence GATGGATTGAGTTTCCCCGA and strand-specific overhangs compatible with ligation after vector digestion with BsmBI. Complementation of *Hoip*$^{-/-}$ cells was achieved by introducing synonymous mutations across the gRNA recognition site in *Hoip* cDNA.

Viral stocks. To purify MNoV, BV2 cultures were frozen and thawed to liberate virions, cleared of cellular debris, then concentrated by filtration through a Vivaflow 200 PES filter

with a 10,000 molecular weight cut-off (Sartorius). Titers were determined by plaque assay on BV2 cells (2, 3). EMCV-D was released from cells by freeze/thaw. VSV, TMEV and EMCV-D were purified by filtration of cell culture supernatant through a 0.22 μm filter. VSV-GFP, TMEV and EMCV-D were titered by plaque assay on Vero, BHK-21, and L929 cells, respectively.

Cell treatments and infections. MEFs were isolated from E14 embryos and transformed with SV40 large T-antigen. Immortalized *Hoil1*$^{f/f}$ MEFs were transiently transfected with a plasmid expressing Cre-recombinase (pTrip-CMV-Cre-Puro-T2A-RFP) using *Trans*IT-LT1 (Mirus), and *Hoil1*$^{-/-}$ and *Hoil1*$^{f/f}$ single-cell clones isolated. To generate *Hoip*$^{-/-}$ MEFs, WT MEFs were transduced with LentiCRISPRv2 expressing gRNA targeting *Hoip* exon 3 and selected with puromycin. Single cell clones were evaluated by immunoblot and sequencing to identify knockout populations.

For lentiviral transductions, HEK293T cells were transfected with lentiviral expression plasmids, along with pMD2.G and psPAX2 lentiviral packaging plasmids, using *Trans*IT-LT1 (Mirus). Supernatants were filtered through a 0.22 μm filter and added to immortalized MEFs. Transduced cells were selected with puromycin or blasticidin.

To assess sensitivity to TNFα-induced cell death, MEFs were plated in tissue-culture treated plates and 20 h later treated with 150 μg/mL TNFα. At 48 h, cells were fixed and stained with 0.1% crystal violet in 20% ethanol for 15 min, then gently washed with water and imaged.

Mitochondrial fractionation. Mitochondrial fractionation was performed using a mitochondria isolation kit (Thermo Scientific) following the manufacturer's protocol with minor modifications. Briefly, six million cells were trypsinized, pelleted, and washed three times with ice-cold PBS. Cell pellets were resuspended in 800 µL of reagent A, vortexed for 5 s, and incubated on ice for 2 min. Cells were lysed using a Dounce homogenizer with 60 strokes. The homogenizer was rinsed with 200 µL of reagent A, which was pooled with the lysate. 800 µL of Reagent C was added to the combined lysate. The lysate was centrifuged at 700× *g* for 10 min at 4°C to pellet cell debris and nuclei. The supernatant was collected and centrifuged at 3,000× *g* for 15 min at 4°C to pellet the mitochondrial fraction. The supernatant (cytosolic fraction) was transferred into a new tube. The mitochondrial pellet was washed twice by resuspension in 500 µL of Reagent C followed by centrifugation at 12,000× *g* for 5 min at 4°C. The mitochondrial pellet was lysed in RIPA buffer supplemented with protease and phosphatase inhibitors. The mitochondrial and cytosolic fractions were denatured with Laemmli sample buffer and heating at 98°C for 10 min.

Semi-denaturing detergent agarose gel electrophoresis (SDD-AGE). MAVS aggregation in MEFs was assessed by SDD–AGE as previously described (4, 5). Briefly, MEFs were infected with mutEMCV (MOI of 0.5) or SeV (10 HAU/mL) for 12 h, or transfected with EMCV-RNA (400 ng/mL) for 16 h. After treatment, cells were harvested and suspended in buffer A (10 mM Tris-HCl, pH 7.5, 1.5 mM $MgCl_2$, 10 mM KCl, 0.25 M D-mannitol), followed by cell lysis through grinding. Cell lysates were centrifuged at 700× *g* for 10 min, and supernatants transferred to a new tube and further centrifuged at

10,000× *g* for 30 min at 4°C to separate the crude mitochondria from cytosolic extracts. Crude mitochondria were resuspended in 1× sample buffer (0.5× TBE, 10% glycerol, 2% SDS, and 0.0025% bromophenol blue) and loaded onto a 1.5% agarose gel. Electrophoresis was performed at 100 V and 4°C until the bromophenol blue dye reached the bottom of the gel. Proteins were transferred onto a PVDF membrane and analyzed by immunoblotting.

IRF3 dimerization. Endogenous IRF3 dimerization was determined by Native PAGE as previously described (6). Briefly, MEFs were infected with mutEMCV (MOI of 5) or transfected with $RABV_{Le}$ RNA (5 pmol/mL) for 8 h, then scraped off in ice-cold PBS, pelleted, and lysed in 60 µL NP-40 buffer containing 10% glycerol. Cell lysates were subject to three freeze-thaw-vortex cycles, followed by centrifugation at 16,000× *g* at 4°C for 20 min. The cleared lysates were mixed with 2× native-PAGE loading buffer and separated on a 7.5% acrylamide/bis-acrylamide (37.5:1) Tris-Glycine gel with 1% sodium deoxycholate added to the inner chamber buffer. Proteins were then transferred onto PVDF membranes and analyzed by immunoblotting.

Immunoblot analyses. Proteins were separated by SDS-PAGE or SDD-AGE and transferred to PVDF membrane. Membranes were incubated with the primary antibodies (**Table S1**) followed by incubation with goat anti-rabbit or anti-mouse IgG-HRP. Blots were incubated with Immobilon Western Chemiluminescent HRP Substrate (Millipore) and imaged on a ChemiDoc Imaging System (Bio-Rad), or using the SuperSignal West Pico PLUS or Femto chemiluminescence reagents (Thermo Fisher Scientific) on an

ImageQuant LAS 4000 Chemiluminescent Image Analyzer (General Electric). In some cases, blots were re-probed with additional antibodies and re-imaged.

Table S1

Target	Company	Product code
HOIL1	Santa Cruz Biotechnologies	sc393754
HOIP	Abcam	ab46322
SHARPIN	Proteintech	14626-I-AP
FLAG	Sigma-Aldrich	M3165
MAVS	Cell Signaling technologies	4983
MAVS	Santa Cruz Biotechnologies	sc365333
MDA5	Adipogen	AL180
LGP2	Proteintech	11355-1-AP
IRF3	Cell Signaling technologies	11904
IRF3 pS396	Cell Signaling technologies	29047
Tom40	Santa Cruz Biotechnologies	sc-365467
HA	Cell Signaling technologies	3724
V5	Cell Signaling technologies	13202
ubiquitin	Santa Cruz Biotechnologies	sc-8017
M1-ubiquitin	Sigma-Aldrich	MABS451
β-actin	Sigma-Aldrich	A5316
GAPDH	Cell Signaling technologies	97166
tubulin	Sigma-Aldrich	T9026

Antibodies used in this study.

Quantification of IFNβ protein in cell supernatants. IFNβ protein in cell supernatants was quantified using a mouse IFN-beta DuoSet ELISA (R&D Systems) according to the manufacturer's instructions.

Supplementary References

1. Sanjana NE, Shalem O, Zhang F. 2014. Improved vectors and genome-wide libraries for CRISPR screening. Nat Methods 11:783-784.
2. Strong DW, Thackray LB, Smith TJ, Virgin HW. 2012. Protruding domain of capsid protein is necessary and sufficient to determine murine norovirus replication and pathogenesis in vivo. J Virol 86:2950-8.
3. Orchard RC, Wilen CB, Doench JG, Baldridge MT, McCune BT, Lee YC, Lee S, Pruett-Miller SM, Nelson CA, Fremont DH, Virgin HW. 2016. Discovery of a proteinaceous cellular receptor for a norovirus. Science 353:933-6.
4. Liu G, Lee JH, Parker ZM, Acharya D, Chiang JJ, van Gent M, Riedl W, Davis-Gardner ME, Wies E, Chiang C, Gack MU. 2021. ISG15-dependent activation of the sensor MDA5 is antagonized by the SARS-CoV-2 papain-like protease to evade host innate immunity. Nat Microbiol 6:467-478.
5. Riedl W, Acharya D, Lee JH, Liu G, Serman T, Chiang C, Chan YK, Diamond MS, Gack MU. 2019. Zika Virus NS3 Mimics a Cellular 14-3-3-Binding Motif to Antagonize RIG-I- and MDA5-Mediated Innate Immunity. Cell Host Microbe 26:493-503 e6.
6. Gack MU, Kirchhofer A, Shin YC, Inn KS, Liang C, Cui S, Myong S, Ha T, Hopfner KP, Jung JU. 2008. Roles of RIG-I N-terminal tandem CARD and splice variant in TRIM25-mediated antiviral signal transduction. Proc Natl Acad Sci U S A 105:16743-8.

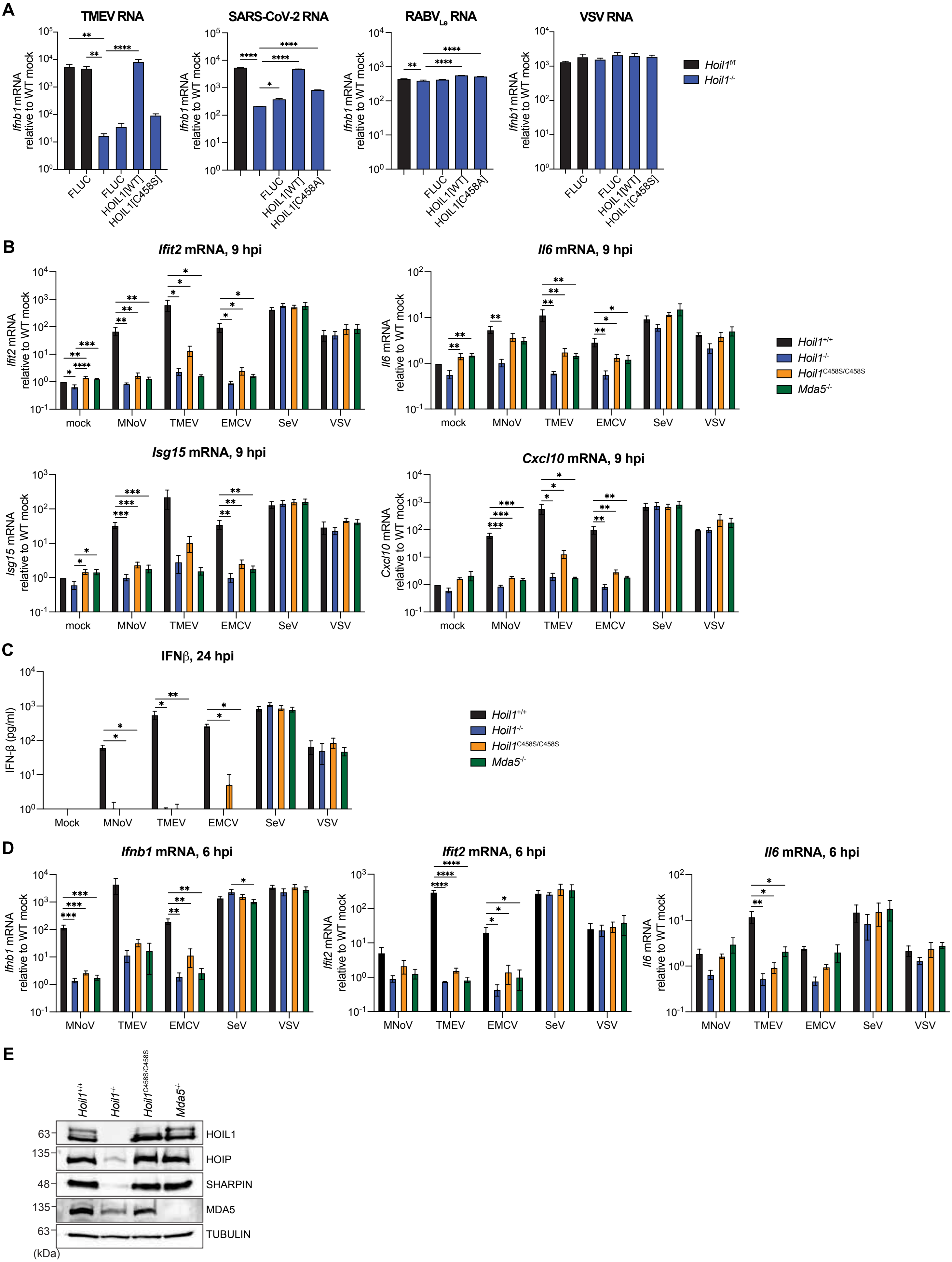
A
TMEV RNA
SARS-CoV-2 RNA
RABV$_{Le}$ RNA
VSV RNA
Ifnb1 mRNA relative to WT mock
FLUC
HOIL1[WT]
HOIL1[C458S]
HOIL1[C458A]
Hoil1$^{f/f}$
Hoil1$^{-/-}$
B
Ifit2 mRNA, 9 hpi
Il6 mRNA, 9 hpi
Isg15 mRNA, 9 hpi
Cxcl10 mRNA, 9 hpi
Ifit2 mRNA relative to WT mock
Il6 mRNA relative to WT mock
Isg15 mRNA relative to WT mock
Cxcl10 mRNA relative to WT mock
mock
MNoV
TMEV
EMCV
SeV
VSV
Hoil1$^{+/+}$
Hoil1$^{-/-}$
Hoil1$^{C458S/C458S}$
Mda5$^{-/-}$
C
IFNβ, 24 hpi
IFN-β (pg/ml)
Mock
D
Ifnb1 mRNA, 6 hpi
Ifit2 mRNA, 6 hpi
Il6 mRNA, 6 hpi
E
HOIL1
HOIP
SHARPIN
MDA5
TUBULIN
63
135
48
(kDa)

Figure S1: HOIL1 ligase activity is required for IRF3- and NF-κB-regulated gene induction in response to MDA5 agonists, but not RIG-I agonists.

(**A**) *Ifnb1* mRNA fold induction in the indicated MEFs after transfection with the indicated viral RNAs: TMEV RNA (500 ng/mL) for 8 h, SARS-CoV-2 RNA (400 ng/mL) for 16 h, $RABV_{Le}$ RNA (2 pmol/mL) for 16 h, or VSV RNA (500 ng/mL) for 8 h. (**B**) Fold induction of *Ifit2*, *Il6, Isg15*, and *Cxcl10* mRNA in WT, *Hoil1*$^{-/-}$, *Hoil1*$^{C458S/C458S}$, and *Mda5*$^{-/-}$ BMDCs at 9 hpi with the indicated viruses, as in Fig. 1F. (**C**) IFNβ protein in BMDC supernatants at 24 hpi with the indicated viruses. (**D**) Fold induction of *Ifnb1, Ifit2* and *Il6* mRNA in WT, *Hoil1*$^{-/-}$, *Hoil1*$^{C458S/C458S}$, and *Mda5*$^{-/-}$ BMDCs at 6 hpi with the indicated viruses. (**E**) Immunoblot analysis of LUBAC subunit and MDA5 expression in uninfected BMDCs. For A, TMEV RNA and VSV RNA, data are combined means from 3-4 independent experiments performed in duplicate. SARS-CoV-2 RNA, and $RABV_{Le}$ RNA data are representative of at least 2 independent experiments. For B and D, data are combined means from 3-5 independent experiments performed in duplicate. For C and E, data are representative of 3 independent experiments. Means ± SEM are shown. Significance was determined by one-way ANOVA compared to *Hoil1*$^{-/-}$ untransduced with Dunnett's multiple comparisons test [A], one-way ANOVA with Tukey's multiple comparisons test [B, C (VSV and SeV), and D], or Kruskal-Wallis test with Dunn's multiple comparison test [C (MNoV, TMEV, EMCV)]. *$p \leq 0.05$, **$p \leq 0.01$, ***$p \leq 0.001$, ****$p \leq 0.0001$.

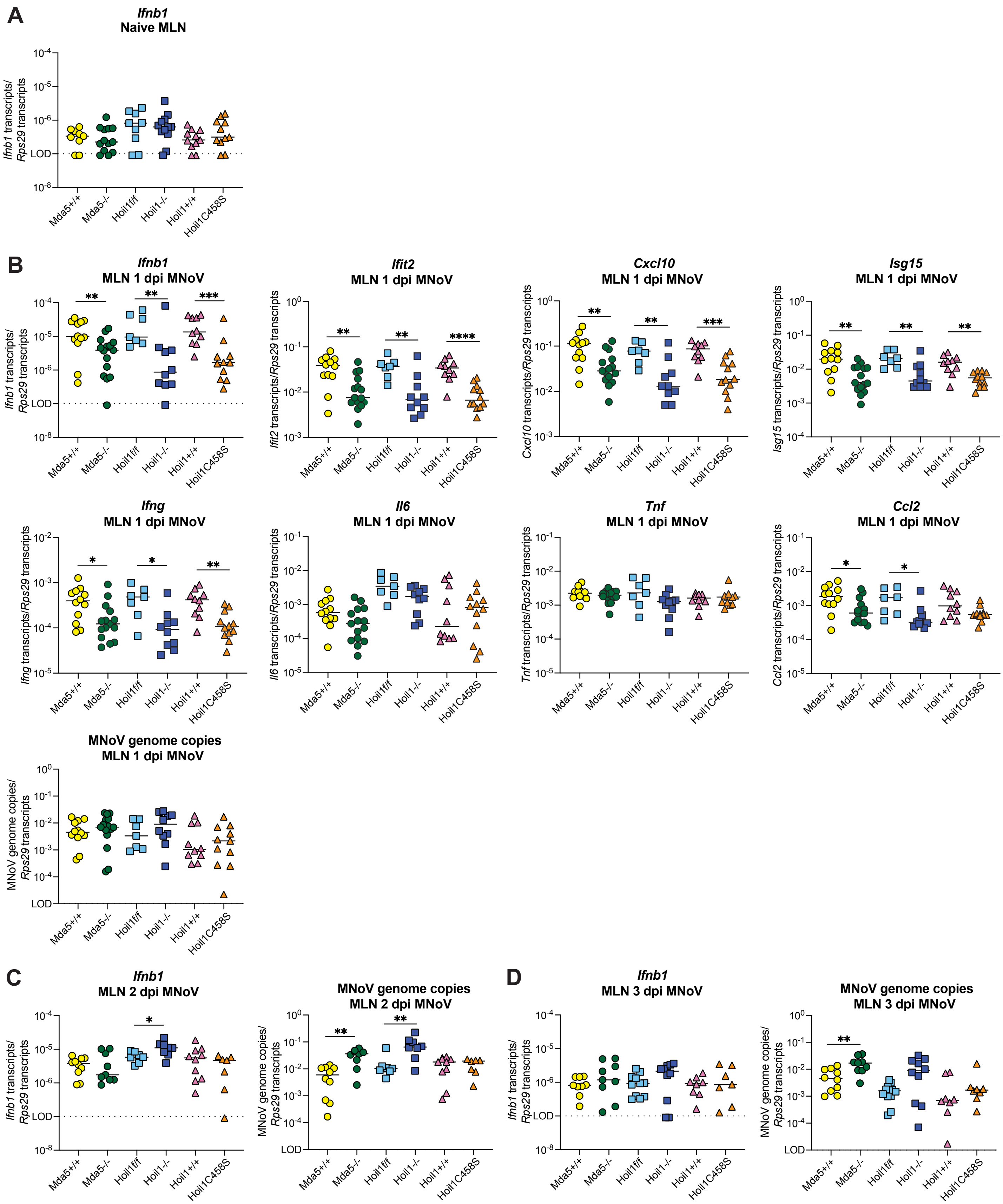
A
Ifnb1
Naive MLN
Ifnb1 transcripts/
Rps29 transcripts
LOD
Mda5+/+
Mda5-/-
Hoil1f/f
Hoil1-/-
Hoil1+/+
Hoil1C458S
B
Ifnb1
MLN 1 dpi MNoV
Ifit2
MLN 1 dpi MNoV
Ifit2 transcripts/Rps29 transcripts
Cxcl10
MLN 1 dpi MNoV
Cxcl10 transcripts/Rps29 transcripts
Isg15
MLN 1 dpi MNoV
Isg15 transcripts/Rps29 transcripts
Ifng
MLN 1 dpi MNoV
Ifng transcripts/Rps29 transcripts
Il6
MLN 1 dpi MNoV
Il6 transcripts/Rps29 transcripts
Tnf
MLN 1 dpi MNoV
Tnf transcripts/Rps29 transcripts
Ccl2
MLN 1 dpi MNoV
Ccl2 transcripts/Rps29 transcripts
MNoV genome copies
MLN 1 dpi MNoV
MNoV genome copies/
Rps29 transcripts
C
Ifnb1
MLN 2 dpi MNoV
MNoV genome copies
MLN 2 dpi MNoV
D
Ifnb1
MLN 3 dpi MNoV
MNoV genome copies
MLN 3 dpi MNoV

Figure S2: HOIL1 E3 ligase is required for the early IFN response during acute MNoV infection *in vivo*. (**A**) *Ifnb1* mRNA expression in MLN from naïve mice. (**B**) *Ifnb1*, *Ifit2*, *Cxcl10*, *Isg15*, *Ifng*, *Il6*, *Tnf*, and *Ccl2* mRNA expression and MNoV genome copies measured in MLN from the indicated mice at 1dpi with 10^6 PFU MNoV CW3 i.g. (**C-D**) *Ifnb1* mRNA expression and MNoV genome copies measured in MLN from the indicated mice at 2 dpi (C) and 3 dpi (D) with 10^6 PFU MNoV CW3 i.g. mRNA and MNoV genome copies were normalized to *Rps29* mRNA copies. Statistical analyses were performed using Mann-Whitney tests between matched groups. **p*≤0.05, ***p*≤0.01, ****p*≤0.001.

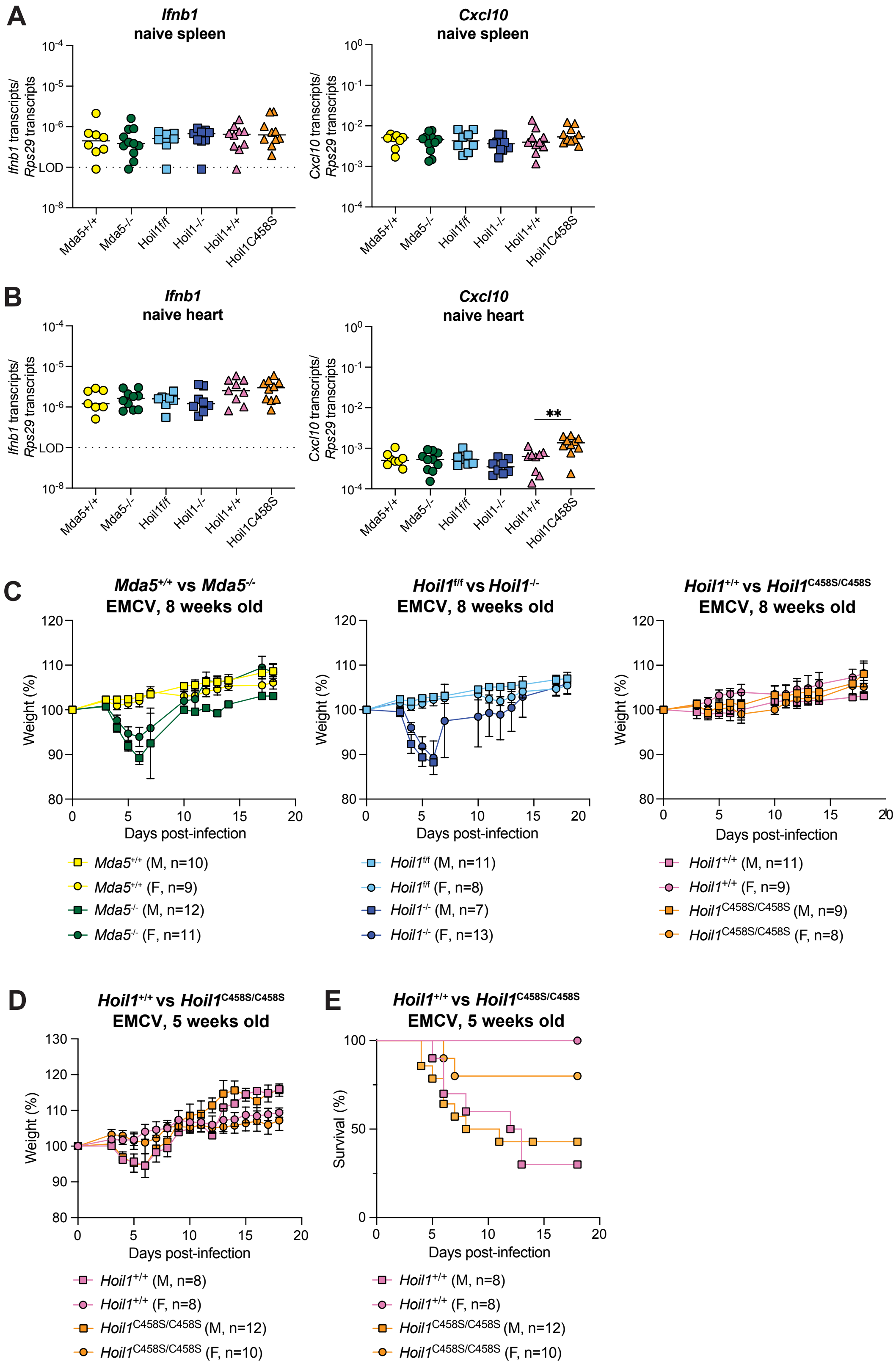

A
Ifnb1
naive spleen
Ifnb1 transcripts/
Rps29 transcripts
LOD
Cxcl10
naive spleen
Cxcl10 transcripts/
Rps29 transcripts
Mda5+/+
Mda5-/-
Hoil1f/f
Hoil1-/-
Hoil1+/+
Hoil1C458S
B
Ifnb1
naive heart
Cxcl10
naive heart
**
C
Mda5+/+ vs Mda5-/-
EMCV, 8 weeks old
Hoil1f/f vs Hoil1-/-
EMCV, 8 weeks old
Hoil1+/+ vs Hoil1C458S/C458S
EMCV, 8 weeks old
Weight (%)
Days post-infection
Mda5+/+ (M, n=10)
Mda5+/+ (F, n=9)
Mda5-/- (M, n=12)
Mda5-/- (F, n=11)
Hoil1f/f (M, n=11)
Hoil1f/f (F, n=8)
Hoil1-/- (M, n=7)
Hoil1-/- (F, n=13)
Hoil1+/+ (M, n=11)
Hoil1+/+ (F, n=9)
Hoil1C458S/C458S (M, n=9)
Hoil1C458S/C458S (F, n=8)
D
Hoil1+/+ vs Hoil1C458S/C458S
EMCV, 5 weeks old
Weight (%)
Days post-infection
Hoil1+/+ (M, n=8)
Hoil1+/+ (F, n=8)
Hoil1C458S/C458S (M, n=12)
Hoil1C458S/C458S (F, n=10)
E
Hoil1+/+ vs Hoil1C458S/C458S
EMCV, 5 weeks old
Survival (%)
Days post-infection
Hoil1+/+ (M, n=8)
Hoil1+/+ (F, n=8)
Hoil1C458S/C458S (M, n=12)
Hoil1C458S/C458S (F, n=10)

Figure S3: Responses to EMCV-D infection in *Mda5*$^{-/-}$, *Hoil1*$^{-/-}$, and *Hoil1*$^{C458S/C458S}$ mice and WT controls. (**A-B**) *Ifnb1* and *Cxcl10* mRNA expression in the spleen (A) and heart (B) from 8-week-old, naïve *Mda5*$^{+/+}$, *Mda5*$^{-/-}$, *Hoil1*$^{f/f}$, *Hoil1*$^{-/-}$, *Hoil1*$^{+/+}$ and *Hoil1*$^{C458S/C458S}$ mice. (**C**) Percent body weight of 8-week-old male (M) and female (F) *Mda5*$^{-/-}$, *Hoil1*$^{-/-}$, and *Hoil1*$^{C458S/C458S}$ mice and WT littermate controls over 18 d with 5000 PFU EMCV-D. (**D-E**) Percent body weight (D) and survival (E) of 5-week-old *Hoil1*$^{C458S/C458S}$ mice and WT littermate controls over 18 d with 5000 PFU EMCV-D. For A and B, male and female mice were included in all groups, each symbol represents an individual mouse, and the median is indicated. For C and D, means ± SEM are shown. Statistical analyses were performed using Mann-Whitney tests between matched groups (A and B) or Log-rank (Mantel-Cox) tests (E). $^{**}p \leq 0.01$.

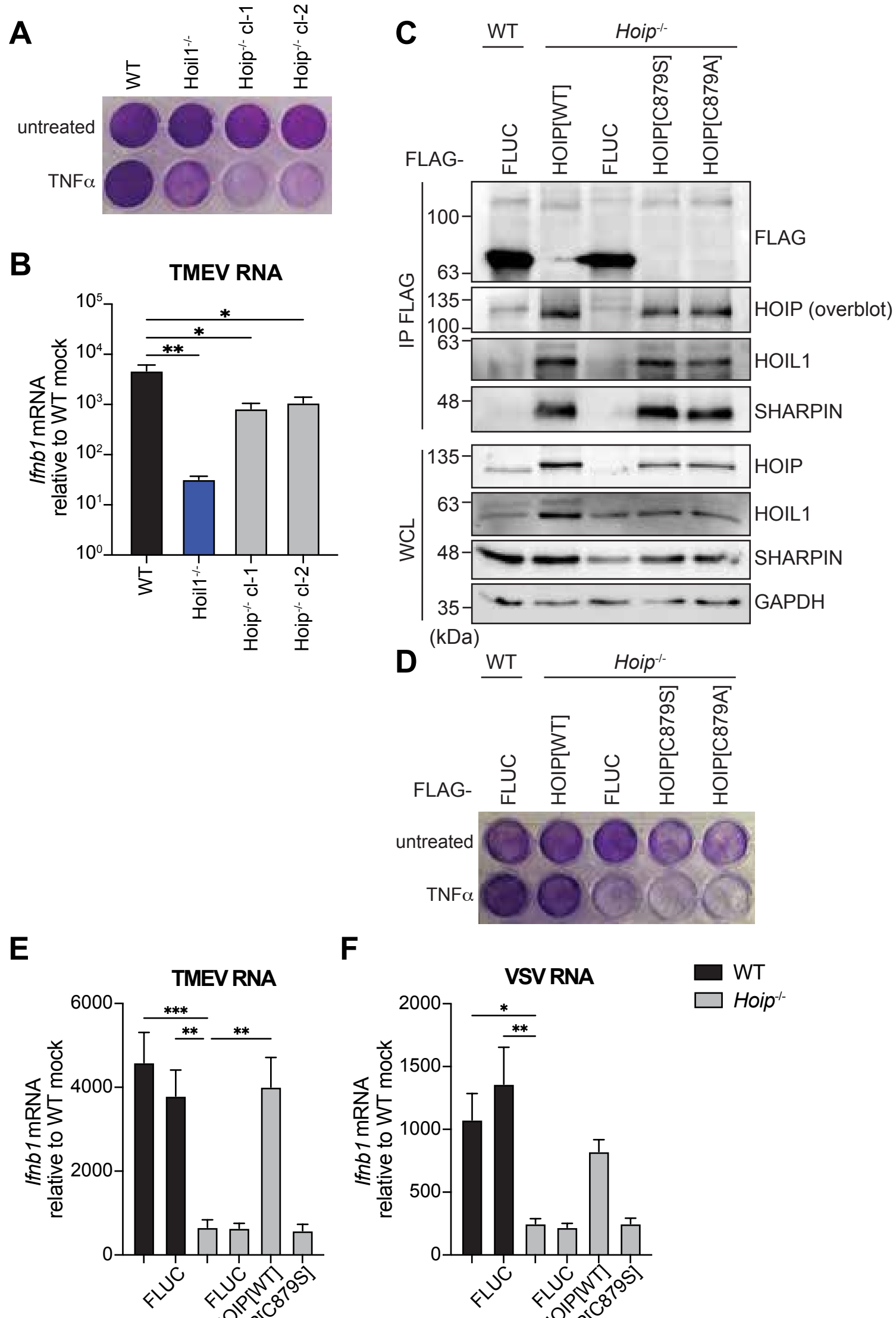
A
WT
Hoil1-/-
Hoip-/- cl-1
Hoip-/- cl-2
untreated
TNFα
B
TMEV RNA
Ifnb1 mRNA relative to WT mock
WT
Hoil1-/-
Hoip-/- cl-1
Hoip-/- cl-2
C
WT
Hoip-/-
FLAG-
FLUC
HOIP[WT]
FLUC
HOIP[C879S]
HOIP[C879A]
IP FLAG
FLAG
HOIP (overblot)
HOIL1
SHARPIN
WCL
HOIP
HOIL1
SHARPIN
GAPDH
(kDa)
D
WT
Hoip-/-
FLAG-
FLUC
HOIP[WT]
FLUC
HOIP[C879S]
HOIP[C879A]
untreated
TNFα
E
TMEV RNA
Ifnb1 mRNA relative to WT mock
FLUC
FLUC
HOIP[WT]
HOIP[C879S]
F
VSV RNA
Ifnb1 mRNA relative to WT mock
FLUC
FLUC
HOIP[WT]
HOIP[C879S]
WT
Hoip-/-

Figure S4: HOIP E3 ligase activity promotes both MDA5- and RIG-I-dependent *Ifnb1* induction. (**A**) Crystal violet-stained TNFα sensitivity assay (150 μg/mL for 48 h) for WT, *Hoil1*$^{-/-}$, *Hoip*$^{-/-}$ clone-1, and *Hoip*$^{-/-}$ clone-2 MEFs. (**B**) *Ifnb1* mRNA fold induction after TMEV RNA transfection for the indicated MEFs. (**C**) Immunoblot analyses of LUBAC subunits in anti-FLAG co-IPs and whole cell lysates from WT and *Hoip*$^{-/-}$ MEFs stably transduced with vectors expressing 3xFLAG-HOIP[WT], -HOIP[C879S], -HOIP[C879A], or -FLUC. (**D**) Crystal violet-stained TNFα sensitivity assay (150 μg/mL for 48 h) for the indicated MEF lines. (**E-F**) *Ifnb1* mRNA fold induction in the indicated MEFs after transfection with the TMEV RNA (E) or VSV RNA (F) (500 ng/mL) for 8 h. For B, E, and F, data are combined from 3-4 independent experiments performed in duplicate, and the mean $\pm$ SEM is shown. For A, C and D, data shown are representative of 3 independent experiments. Significance was determined by one-way ANOVA with Tukey's multiple comparisons test (B), or by one-way ANOVA compared to *Hoip*$^{-/-}$ untransduced with Dunnett's multiple comparisons test (E and F). *$p \leq 0.05$, **$p \leq 0.01$, ***$p \leq 0.001$.

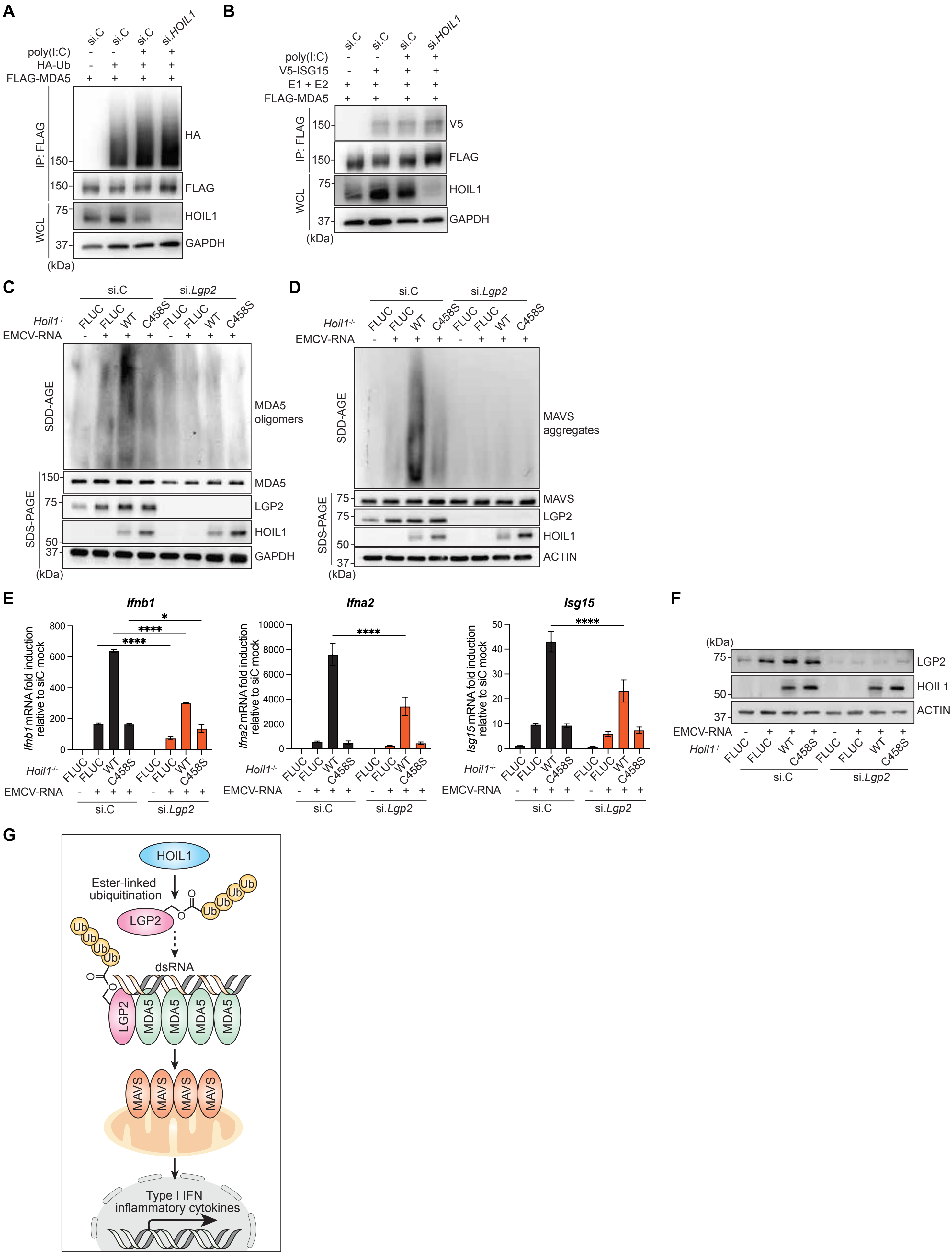
A
si.C
si.C
si.C
si.HOIL1
poly(I:C)
HA-Ub
FLAG-MDA5
IP: FLAG
HA
FLAG
WCL
HOIL1
GAPDH
(kDa)
B
poly(I:C)
V5-ISG15
E1 + E2
FLAG-MDA5
V5
FLAG
HOIL1
GAPDH
C
si.C
si.Lgp2
Hoil1-/-
FLUC
WT
C458S
EMCV-RNA
SDD-AGE
MDA5 oligomers
SDS-PAGE
MDA5
LGP2
HOIL1
GAPDH
D
MAVS aggregates
MAVS
LGP2
HOIL1
ACTIN
E
Ifnb1
Ifnb1 mRNA fold induction relative to siC mock
Ifna2
Ifna2 mRNA fold induction relative to siC mock
Isg15
Isg15 mRNA fold induction relative to siC mock
F
(kDa)
LGP2
HOIL1
ACTIN
G
HOIL1
Ester-linked ubiquitination
LGP2
Ub
dsRNA
MDA5
MAVS
Type I IFN
inflammatory cytokines

Figure S5: HOIL1 and LGP2 both function upstream of MAVS to regulate MDA5 signaling. (**A-B**) Ubiquitination (HA-Ub, A) or ISGylation (V5-ISG15, B) of FLAG-hMDA5 immunoprecipitated from HEK293T cells after siRNA-mediated knock-down (KD) of *HOIL1* (si. *HOIL1*) or control (si.C) and stimulation with poly(I:C) (1 μg/mL) for 6 h, or unstimulated. Expression of HOIL1 is indicated in the whole cell lysates (WCL). (**C-D**) SDD-AGE immunoblot analyses of MDA5 oligomers (C) and MAVS aggregates (D) induced by EMCV-RNA (400 ng/mL for 16 h) in *Hoil1*$^{-/-}$ MEFs complemented with FLUC, HOIL1[WT] or HOIL1[C458S], after control (si.C) or *Lgp2* (si.*Lgp2*) KD. Total protein expressions are shown by SDS-PAGE immunoblots below. (**E**) *Ifnb1*, *Ifna2*, and *Isg15* mRNA induced by EMCV-RNA (400 ng/mL for 16 h) in the indicated MEF lines after si.C or si.*Lgp2* transfection. (**F**) Immunoblot analysis confirming LGP2 KD for the experiment shown in E. (**G**) Model illustrating HOIL1's role in MDA5 signaling. HOIL1 E3 ligase ubiquitinates LGP2 and promotes MDA5 oligomer and MAVS aggregate formation. Data shown are representative of at least two independent experiments. Statistical analyses were performed using unpaired two-way ANOVA with Šidák's multiple comparisons test. *$p \leq 0.05$, ****$p \leq 0.0001$.
